# FGFR inhibition as a new therapeutic strategy to sensitize glioblastoma stem cells to tumor treating fields

**DOI:** 10.1038/s41420-025-02542-5

**Published:** 2025-06-04

**Authors:** Pauline Deshors, Ziad Kheil, Laetitia Ligat, Valerie Gouazé-Andersson, Elizabeth Cohen-Jonathan Moyal

**Affiliations:** 1 Oncopole Claudius Regaud, IUCT-Oncopole Toulouse, Toulouse, France; 2 INSERM U1037, Cancer Research Center of Toulouse (CRCT), Toulouse, France; 3 University Toulouse, Toulouse, France

**Keywords:** Cancer stem cells, Cancer stem cells, Cancer therapeutic resistance

## Abstract

Glioblastomas (GBM) are aggressive tumors, which systematically relapse despite standard treatment associating surgery, chemotherapy and radiation therapy. More recently, GBM therapy now includes another therapeutic modality option, Tumor Treating Fields (TTFields) given in combination with Temozolomide (TMZ) following standard treatment. However even with the adjunction of TTFields, GBM remains a lethal disease due to treatment resistance. One of the causes of resistance is the presence of cancer stem cells (GSC) known to be chemo and radioresistant and responsible for tumor regrowth. Studying mechanisms of resistance of GSC to TTFields is thus a major issue to address. Fibroblast Growth Factor Receptors (FGFR) play a major role in numerous processes essential for cancer development, and dysregulation of FGFR signaling has been observed in many cancer types, including GBM. We have previously shown that tyrosine kinase receptor Fibroblast Growth Factor Receptor 1 (FGFR1) controls GBM aggressiveness and GSC radioresistance and that its inhibition leads to radiosensitization through increasing mitotic cell death and microenvironment modulation. Because one of the main mechanisms of action of TTFields is mitotic disturbance and because TTFields act synergistically in vitro with irradiation (IR), we hypothesize that targeting FGFR could sensitize GSC to TTFields. Here we show that, like IR, TTFields significantly decrease GSC growth. Treatment of GSC with pemigatinib (Pem), a FGFR1-3 inhibitor, alters FGFR signalling pathway. We demonstrate that Pem, sensitizes GSC to TTFields by synergistically decreasing their survival and clonogenic ability. Finally, the adjunction of Pem to treatment combining IR and TTFields could sensitize GSC by inducing, in some GSC, a further decrease in the repair of IR-induced DNA damages. Altogether, these results highlight the potential benefits of inhibiting FGFR with the concomitant application of TTFields in the first-line standard GBM treatment to improve patient prognosis.

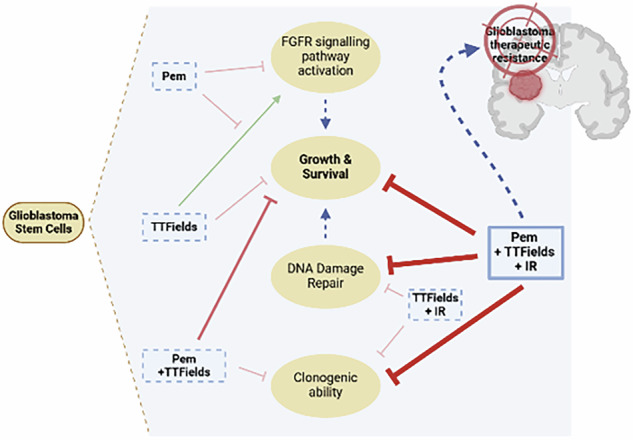

## Introduction

Glioblastoma (GBM) is the most aggressive and frequent primary malignant brain tumour in adults accounting for 48.6% of malignant central nervous system tumours and has an incidence about 4/100,000 per year [1]. They are classified as grade-IV cancers according to the World Health Organization (WHO). Despite conventional treatment associating surgical resection followed by ionizing radiations (IR) and concomitant and adjuvant chemotherapy with Temozolomide (TMZ), these tumours display a poor prognosis and patients inevitably recur [2, 3]. Only few therapeutic advances have been made for GBM treatment over the past decades but, Tumour Treating Fields (TTFields) achieved improved outcomes in clinical trials by increasing patients median overall survival from 15 to 20.9 months [4, 5]. TTFields are now a part of the first-line treatment for newly diagnosed GBM and malignant pleural mesothelioma (MPM), with ongoing clinical trials in multiple other solid tumours [4–7]. This non-invasive, physical, local therapy relies on the delivery, *via* pairs of electrode arrays placed on the patient's skin at the tumour site, of alternating electrical fields with low intensity (1-3V/cm) and intermediate frequency (100 to 500kHz, depending on the tumour type). They target cancer cells by disturbing the localization and polymerization of polar molecules thus altering a multitude of biological processes. In fact, they mainly disrupt cancer cell mitosis [8, 9], but also perturb cell invasion, migration, autophagy, DNA damage response and repair, immune response and angiogenesis [10–18]. Interestingly, due to their multimechanistic actions, TTFields have been described as acting additively or synergistically with other cancer treatments. Preclinical data in multiple tumour models show enhanced effects when TTFields are used concomitantly with chemotherapy, including PARP inhibitors (Poly ADP Ribose Polymerase inhibitors) [15, 16, 19–22] and immune checkpoint inhibitors [12, 21]. In GBM, preclinical and clinical studies have shown that TTFields increase the effectiveness of TMZ and lomustine [18–20, 22, 23]. TTFields also act synergistically with IR regardless the sequence in which they are applied [10, 15, 16, 24]. In fact, as TTFields, IR also induces DNA damage, apoptosis, autophagy, Reactive Oxygen Species (ROS) production and immune response [25]. Based on these preclinical data and a pilot study showing that TTFields concomitant with IR and TMZ is well tolerated [26], the EF-32 international phase III randomized trial (TRIDENT - NCT04471844) was designed and is ongoing to compare the efficacy of standard IR plus TMZ with the combination of IR, TMZ and concomitant TTFields in newly diagnosed GBM [27].

However, although TTFields increase patient’s survival [4], GBM is still an incurable disease with recurrences due to treatment resistance. Therefore, it is crucial to understand mechanisms of GBM resistance to TTFields in order to identify new therapeutic targets.

It is now admitted that GBM resistance and recurrence are linked to the presence of a subpopulation of tumour stem cells, highly chemo- and radio-resistant. These cells, called GBM Stem Cells (GSC), are characterized by their high expression of stem cells markers (i.e. Olig2, Nestin, Sox2, Nanog), their ability to self-renew in vitro and in vivo, and their high tumorigenic potential in vivo [28–30]. The therapeutic resistance of GSC, as well as their ability to maintain the tumour, make them key targets to find more effective therapies for GBM.

Fibroblast Growth Factor Receptors (FGFR), a family of four tyrosine kinase receptors (FGFR1-4), have emerged as potential cancer targets, since FGFR alterations (gene amplifications, mutations, rearrangements/fusions) contribute to tumour development and progression. In glioma, FGFR abnormalities occur in approximately 8% of tumours, mainly affecting FGFR1 and FGFR3 [31, 32]. Upon FGF (Fibroblast Growth Factor) ligand binding, FGFR dimerize and activate via autophosphorylation of cytoplasmic tyrosine residues [31], thus regulating proliferation, survival, differentiation, migration and angiogenesis by activation of downstream signalling pathways. Expression and roles of FGFR1-4 in GBM are heterogeneous, with FGFR1 being the most extensively studied, with a key role in GSC maintenance, proliferation and GBM therapeutic resistance [33–36]. We previously showed that FGFR1 expression correlates with poor clinical outcomes in GBM [37]. Moreover, FGFR1 expression in glioma is linked to increased cell migration [38], and FGFR1 inhibition reduces tumour growth and GSC migration [33, 36]. Moreover, FGFR1 is enriched in GSC and promotes tumorigenicity in vivo [39]. Our group has also shown that FGFR1 controls GBM cells and GSC radioresistance and that its inhibition sensitizes cells to radiation both in vitro and in vivo by increasing mitotic cell death and microenvironment modulation [34, 35, 40]. In contrast, FGFR2 exhibits reduced expression with increasing glioma grade and correlating with poor prognosis [41]. However, FGFR2 inhibition reduces tumour growth, indicating a moderating role in glioma progression [42]. Conversely, FGFR3 is highly expressed in invasive GBM cells, suggesting a role in tumour invasion, although the underlying mechanisms remain unclear [43]. In some gliomas, oncogenic FGFR3-TACC3 (Transforming Acidic Coiled-Coil containing proteins) or FGFR1-TACC1 fusions cause constitutive FGFR3 or FGFR1 activation, promoting tumour growth [44, 45]. FGFR4 expression in GBM is generally low and poorly documented, with one study linking it to higher-grade astrocytomas, though its role remains unclear [46].

Given the pivotal role of FGF signaling in cancer and its influence on therapeutic responses, particularly to IR, alongside our previous findings, and the shared mechanisms between TTFields and IR, we hypothesize that targeting FGFR could enhance GSC sensitivity to TTFields. Here, we report that inhibiting FGFR with pemigatinib (Pem), a potent and selective FGFR1-3 inhibitor, significantly reduces GSC survival after TTFields. Moreover, GSC survival is further decreased when Pem is combined with TTFields and IR, strongly suggesting that FGFR inhibition in combination with standard treatment such as radiotherapy and TTFields could improve their efficacy for patients with GBM.

## Results

### TTFields and IR reduce GSC growth and clonogenicity

**Fig. 1:**
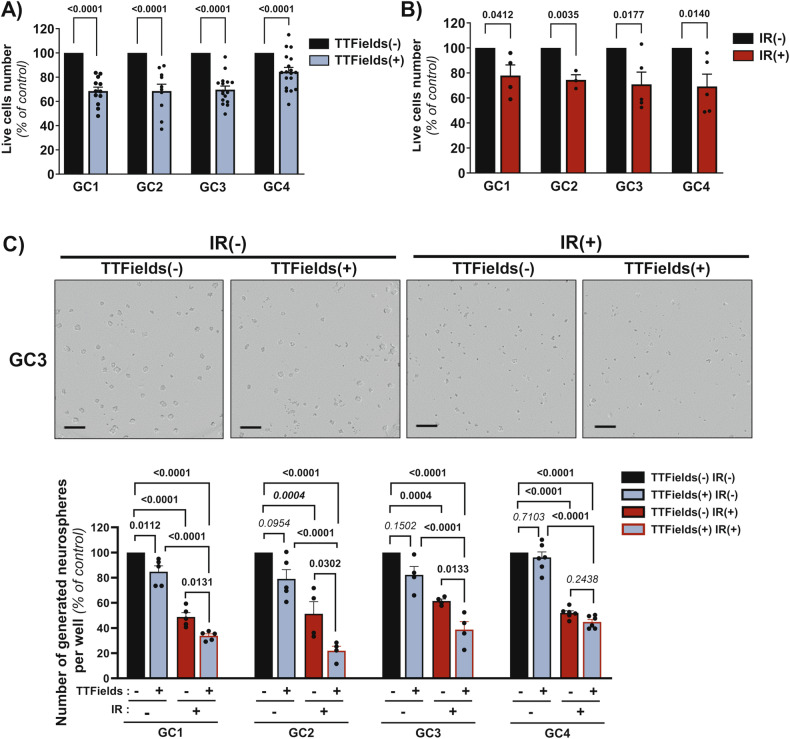
Response of GSC to TTFields and IR treatment alone or in combination. **A** Percentage of live cells per dish determined by cell count after 72 h of TTFields at 200kHz (TTFields(+)). Results are normalized to untreated cells (TTFields(-)). Errors bars show means±SEM of N>10 experiments. **B** Percentage of live cells determined by cell count 72 h after 4Gy irradiation (IR(+)). Results are normalized to untreated cells (IR(-)). Errors bars show means±SEM of N≥3 experiments. **C** Clonogenic assay with GSC treated by TTFields followed by IR. After TTFields treatment (TTFields(+)) or no treatment (TTFields(-)), dissociated GSC were seeded in 96-well plates, exposed to radiation at a dose of 4Gy (IR(+)) or not irradiated, as control (IR(-)), and incubated for 7days in complete medium at 37 °C. The number of neurospheres was then counted in each well. Graphs represent the means ± SEM of the percentage of formed neurospheres per well (normalized to TTFields-untreated, non-irradiated condition (TTFields(-), IR(-)). Errors bars show means±SEM of *N* ≥ 4 experiments. Scale bars 500 µm.

A panel of primary GSC was established from patient’s surgical GBM samples. We did not notice any significant difference in the proliferative capacity among the different GSC tested (Supplementary Fig. 1). To evaluate the sensitivity of the cells to TTFields, they were treated with TTFields at 200 kHz (the GBM treatment frequency) for 72 h, and their viability was assessed by cell counting. TTFields treatment significantly decreased the viability by 15–30% of every tested GSC (Fig. 1A). We also evaluated the response of these GSC to IR at 4 Gy and observed a significant reduction in GSC survival 72 h after IR (Fig. 1B). As TTFields treatment synergises with IR [16, 24], we next analysed the effect of applying TTFields followed by IR at 4Gy (TTFields>IR) on GSC clonogenic ability. TTFields alone, slightly reduced GSC sphere formation. As expected, TTFields followed by IR significantly reduced the number of spheres formed per well compared to either IR or TTFields alone in GC1-3 (Fig. 1C). Although not statistically significant, a similar trend was observed in GC4 (Fig. 1C). Moreover, the effect of the concomitant application of IR and TTFields on GSC clonogenic ability was independent of the treatment sequence since TTFields after IR (IR>TTFields) also significantly decreased the number of spheres (Supplementary Fig. 2). To better characterize the interaction between TTFields and IR, we performed BLISS analysis [47]. We identified a synergistic effect between IR and TTFields in all GSC except for GC4, where an additive effect was observed (Supplementary Table 1). These results indicate at least an additive effect between IR and TTFields. We hypothesized that identifying common targets between IR and TTFields could enhance GSC sensitivity to both therapies.

### TTFields increase FGFR signalling pathway activation in GSC

**Fig. 2:**
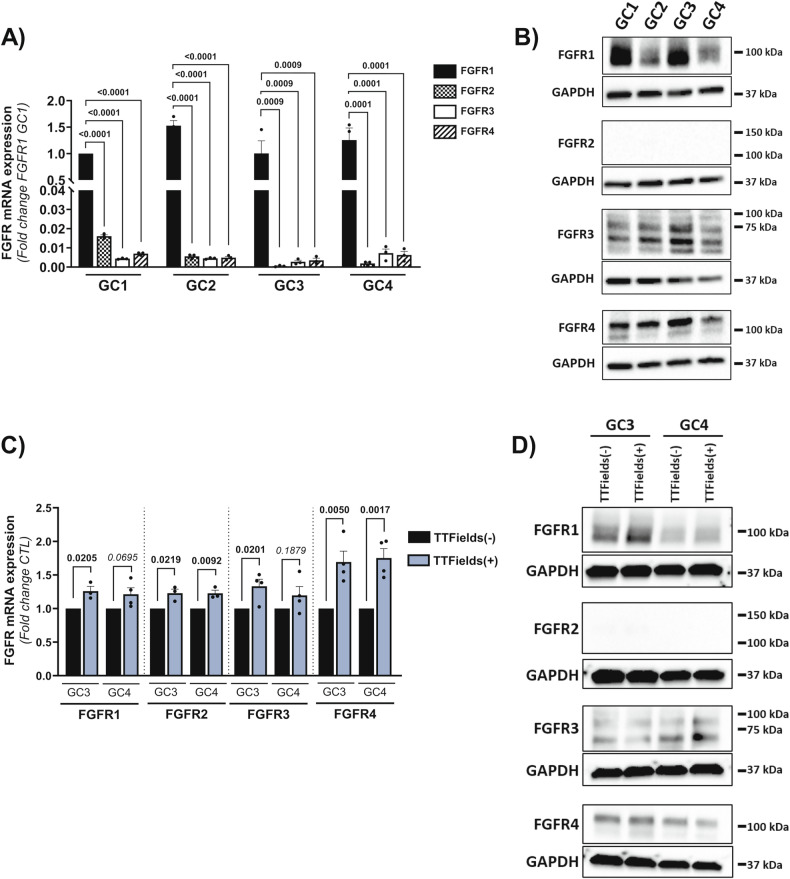
FGFR1-4 expression in GSC in response to TTFields. **A** Relative RNA expression of FGFR1, FGFR2, FGFR3 and FGFR4 determined by Real-time PCR and expressed as Fold change in GSC compared to FGFR1 expression in GC1. Errors bars show means ± SEM of *N* = 3 experiments. **B** Immunoblots of FGFR1, FGFR2, FGFR3 and FGFR4 proteins in GSC. GAPDH was used as loading control. **C** Relative RNA expression of FGFR1, FGFR2, FGFR3 and FGFR4 determined by Real-time PCR and expressed as Fold change in GC3 and GC4 treated by TTFields (TTFields(+)) compared to control without treatment (TTFields(-)). Errors bas show means ± SEM of *N* ≥ 3 experiments. **D** Immunoblots of FGFR1, FGFR2, FGFR3 and FGFR4 proteins in GC3 and GC4 treated with TTFields (TTFields(+)) or untreated (TTFields(-)). GAPDH was used as loading control.

**Fig. 3:**
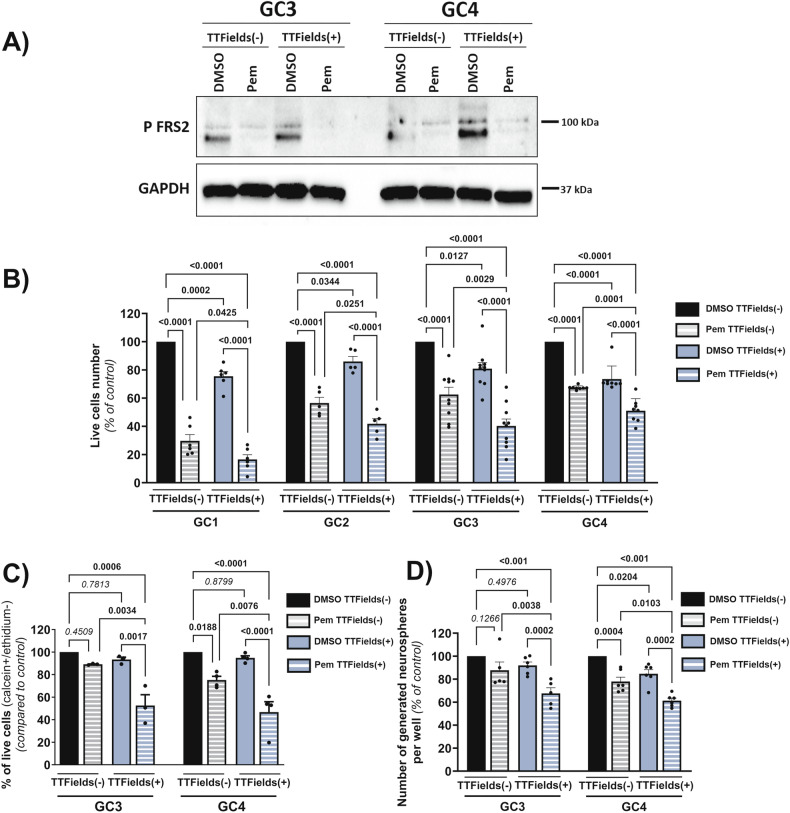
Pem treatment sensitizes GSC to TTFields. **A** Immunoblot of phosphorylated FRS2 (Tyr196) in GC3 and GC4 treated with TTFields (TTFields(+)) or untreated (TTFields(-)) and treated with Pem (Pem) or DMSO as control (DMSO). GAPDH was used as loading control. **B** Percentage of live cells per dish obtained by cell count after 72 h of TTFields (TTFields(+)) or no treatment (TTFields(-)) combined with treatment with Pem (Pem) or DMSO as control (DMSO) in GC1, GC2, GC3 and GC4 cells. Results are normalized to TTFields-untreated condition treated with DMSO for each cell line (DMSO TTFields(-)). Errors bars show means ± SEM of *N* ≥ 5 experiments. **C** Percentage of live cells determined by flow cytometry. Graph represents means ± SEM of the percentage of cells positive for calcein (calcein+) and negative for ethidium staining (ethidium-) in GC3 and GC4 treated with TTFields for 72 h (TTFields(+)) or untreated (TTFields(-)) combined with treatment with Pem (Pem) or DMSO as control (DMSO). Results are normalized to TTFields-untreated condition treated with DMSO for each cell line (DMSO TTFields(-)). Errors bars show means±SEM of *N* ≥ 3 experiments. **D** Clonogenic assay with GC3 and GC4 treated with TTFields (TTFields(+)) or un-treated (TTFields(-)) for 72 h in combination with Pem treatment (Pem) or DMSO as control (DMSO). Dissociated GSC were seeded in 96-well plates and incubated for 7days in complete medium at 37 °C supplemented with Pem (Pem) or DMSO as control (DMSO). The number of neurosphere was then counted in each well. Graphs represent the means ± SEM of the percentage of formed neurospheres per well (normalized to TTFields-untreated and DMSO-treated cells (DMSO TTFields(-)). Errors bars show means ± SEM of *N* ≥ 5 experiments.

As FGFR, and particularly FGFR1, are key regulators of GSC maintenance, radiation resistance and glioma proliferation [33–36], and because TTFields affect tumour cell proliferation, we hypothesized that FGFR might be involved in GSC response to TTFields. FGFR1 mRNA is significantly more expressed in our cells compared to FGFR2-4 (Fig. 2A). All GSC express FGFR1, FGFR3 and FGFR4 proteins, but we did not detect FGFR2 protein in any of the GSC (Fig. 2B). Interestingly, expression levels, particularly of FGFR1, varied across GSC (Fig. 2B). We then analysed the impact of TTFields on FGFR expression in GC3 and GC4. TTFields increased mRNA expression of all four FGFR (Fig. 2C). At the protein level, TTFields application enhanced only FGFR1 expression, with no changes in others FGFR (Fig. 2D and Supplementary Fig. 3). We then investigated the influence of TTFields on FGFR signalling pathway activation. TTFields increased the phosphorylation of FRS2 (FGFR Substrate 2), a downstream effector of FGFR, confirming FGFR signalling activation induced by TTFields (Fig. 3A). FGFR inhibition could therefore be a promising strategy to sensitize GSC to TTFields, similar to IR.

### Pem treatment decreases GSC survival after TTFields

To confirm that targeting FGFR could increase GSC response to TTFields, cells were treated with pemigatinib (Pem), a potent and selective FGFR1-3 inhibitor. Treatment of GSC with 125nM of Pem highly decreased phosphorylation of FRS2 (Fig. 3A), thus confirming the inhibitory effect of Pem on FGFR signalling in these conditions. The increase in FRS2 phosphorylation after TTFields was also inhibited by Pem (Fig. 3A). We then assessed the effect of the concomitant application of Pem and TTFields (TTFields+Pem) on GSC growth. Cell growth of GSC treated with TTFields+Pem, was significantly reduced compared to treatment with TTFields alone or Pem alone (Fig. 3B). To confirm the direct impact of TTFields+Pem on cell survival, we quantified the percentage of live and dead cells in our cultures. As shown in Fig. 3C, TTFields+Pem induced a greater decrease in cell survival, associated to an increase in cell death, than treatment with Pem or TTFields alone (Fig. 3C and Supplementary Fig. 4). Moreover, Pem and TTFields act synergistically to reduce GSC survival (Supplementary table 2). We also evaluated the effect of the combination TTFields+Pem on GSC clonogenic capacity. As shown in Fig. 3D, sphere formation was significantly reduced when Pem and TTFields are combined compared to their separated application (Fig. 3D). Again, Bliss Index indicates that Pem and TTFields acts synergistically to reduce GSC sphere formation (Supplementary Table 3). These data demonstrated that the association of Pem and TTFields synergistically reduces GSC survival and clonogenic capacity and that the concomitant application of TTFields+Pem might be an interesting therapeutic modality.

### Combination of Pem, IR and TTFields decreases GSC survival partially through a disruption of DNA damage repair

**Fig. 4:**
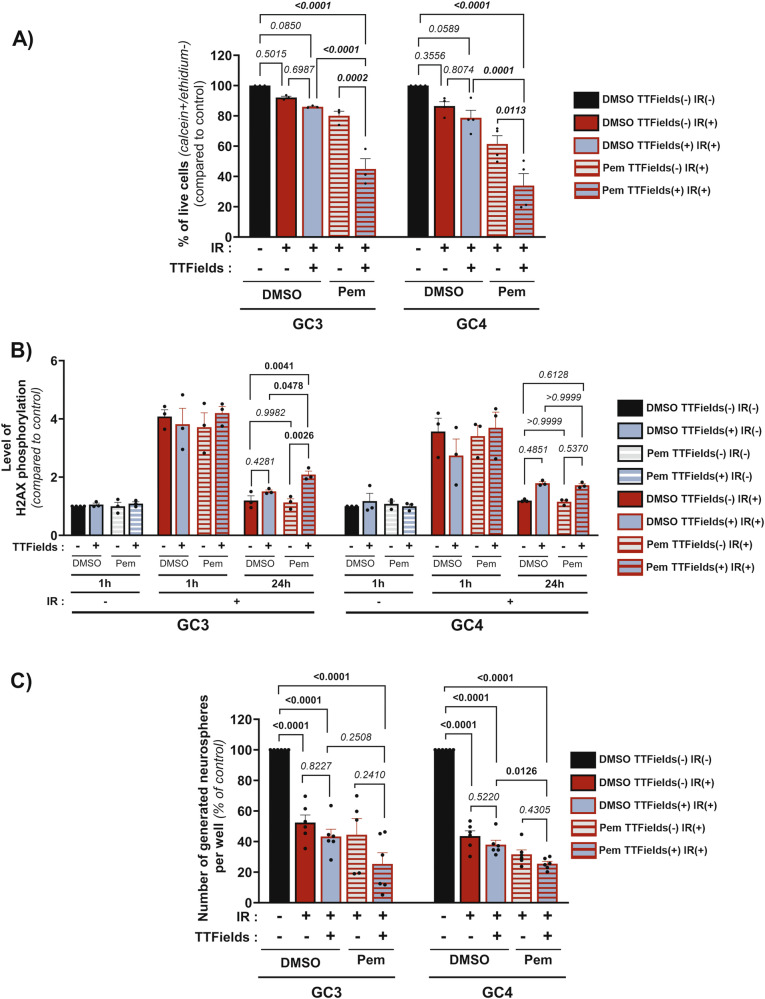
Pem sensitizes GSC to combination of TTFields and IR through alteration of DNA damage repair and reduction in clonogenic capacity. **A** Percentage of live cells determined by flow cytometry. Graph represents means ± SEM of the percentage of cells positive for calcein (calcein+) and negative for ethidium staining (ethidium-) in GC3 and GC4 irradiated (IR+) or non-irradiated (IR-) and then treated with TTFields for 72 h (TTFields(+)) or not (TTFields(-)) combined with treatment with Pem (Pem) or DMSO as control (DMSO). Results are normalized to non-irradiated, TTFields-untreated condition treated with DMSO for each cell line (DMSO TTFields(-) IR(-)). Errors bars show means±SEM of *N* ≥ 3 experiments. **B** Flow cytometry analysis of expression of phosphorylated (Ser139) H2AX (γH2AX) in GC3 and GC4 irradiated at 4Gy (IR(+)) or non-irradiated (IR(-)) and then treated with TTFields (TTFields(+)) or not (TTFields(-)) combined with treatment with Pem (Pem) or DMSO as control (DMSO) for 1 h or for 24 h. Graph represents means ± SEM of the SFI (Specific Fluorescent Index, see material and methods) in treated GSC (Pem/DMSO, TTFields±, IR±) normalized to untreated GSC (DMSO, TTFields(-), IR(-)) for each cell line. Errors bars show means±SEM of N≥3 experiments. **C** Clonogenic assay with GSC treated with TTFields (TTFields(+)) or un-treated (TTFields(-)) for 72 h in combination with Pem treatment (Pem) or DMSO as control (DMSO). Then, dissociated GSC were seeded in 96-well plates and exposed to radiation at 4Gy (IR(+)) or not irradiated as control (IR(-)) and incubated for 7days in complete medium at 37 °C supplemented with Pem (Pem) or DMSO as control (DMSO). The number of neurospheres was then counted in each well. Graphs represent the means ± SEM of the percentage of formed neurospheres per well (normalized to non-irradiated, TTFields untreated and DMSO-treated cells (DMSO, TTFields(-), IR(-)). Errors bars show means ± SEM of *N* ≥ 4 experiments.

Standard of care for GBM patients includes radio-chemotherapy followed by TTFields. We thus studied the effect of the concomitant application of TTFields+Pem after IR on GSC survival. The application of IR followed by TTFields and Pem (IR>TTFields+Pem) significantly, and synergistically, reduced GSC survival compared to treatment with IR and TTFields (IR>TTFields), as well as to treatment with IR and Pem (IR>Pem) (Fig. 4A and Supplementary Table 4). These results are correlated with an increase in the percentage of dead cells (Supplementary Fig. 5). TTFields synergise with IR to enhance its efficacy by inhibiting the repair of DNA damage induced by IR [10, 15]. Thus, we quantified by flow cytometry, in GC3 and GC4, the influence of the association of TTFields, IR and Pem on the phosphorylation levels of histone variant H2AX (γH2AX), a marker of DNA damage (Fig. 4B). As expected, IR induced an increase in γH2AX after 1 h for every treatment combination in the two primocultures. In control condition, 24 h after IR, level of H2AX phosphorylation fell back to its initial level, meaning that DNA damage repair mechanisms were effective. However, the association of TTFields and IR resulted in more residual phosphorylated H2AX at 24 h. In GC3, when TTFields were associated with Pem administration after IR (IR>TTFields+Pem), there was more residual γH2AX signal compared to TTFields alone (IR>TTFields), meaning that the concomitant application of Pem+TTFields further delays DNA damage resolution (Fig. 4B). This result was not observed in GC4 (Fig. 4B).

It has been described that TTFields and IR also synergize when TTFields are administrated before IR (TTFields>IR). Moreover, TTFields, IR and drugs inducing replication stress were all synergistic when TTFields were followed by IR [16, 24]. Thus, we studied by immunofluorescence the influence of the adjunction of Pem when IR follows TTFields treatment (TTFields+Pem>IR+Pem) on γH2AX foci formation. We did not observe any decrease in the level of γH2AX staining between 1 h and 24 h in GC3 when IR was associated with TTFields+Pem, meaning that the concomitant application of TTFields+Pem blocks the repair of DNA damages induced by IR (Supplementary Fig. 6). In GC4, we did not observe the blockage in the repair of DNA damages induced by IR when TTFields and Pem are applicated concomitantly (Supplementary Fig. 6). Finally, we studied the effect of the association of TTFields, Pem and IR on GSC clonogenicity. The application of TTFields and Pem followed by IR (TTFields+Pem>IR+Pem) reduced, even not significantly, GSC sphere formation compared to treatment with TTFields and IR (TTFields>IR), as well as to treatment with Pem and IR (Pem>IR+Pem) (Fig. 4C). For GC3, a synergistic effect was observed, while in GC4 the effect appears to be additive (Supplementary Table 5). Altogether, these results highlight that Pem sensitizes GSC to TTFields associated to IR by decreasing their survival and clonogenicity. An alteration of DNA damage repair processes could explain the sensitization of certain primocultures to this treatment, independently of the treatment sequence.

## Discussion

For almost a decade, standard of care for GBM patients has been concomitant TMZ chemotherapy and radiotherapy [3] and has not been changed despite several attempts to combine it with targeted drugs as cilengitide [48, 49], bevacizumab [50, 51] or immunotherapy with nivolumab [52, 53]. Although the adjunction of TTFields to GBM treatment has allowed the improvement of patients care [4, 5], patient’s survival is still very low and recurrences inevitably occur. Thus, it is of particular interest to find new therapeutic targets to sensitize GBM to therapies. Therapeutic combination might be the best solution and TTFields have been described as acting synergistically with a plethora of therapeutic modalities [10, 12, 15, 16, 20, 22–24, 54, 55]. We and others previously identified FGFR signalling as a major actor of aggressiveness and therapeutic resistance, particularly FGFR1 in GBM [31, 33–38, 40]. In the present work, we demonstrate that FGFR inhibition is also a significant way to increase TTFields sensitivity. As we already observed with IR [34], we demonstrate an increase of FGFR1 expression in GSC after TTFields application. In this work, we show that TTFields also increase the activation of FGFR signalling confirming its role in the adaptation of GSC to TTFields and the interest in targeting FGFR in order to improve GSC response to TTFields.

These results are of particular interest since therapeutic targeting of FGF and their receptors is a key area in cancer research. In fact, FGFR have a major role in numerous processes essential for cancer development and dysregulation of FGFR signalling has been observed in many cancer types, including GBM. Interestingly, FGFR expression is also correlated to therapeutic resistance [56] and FGFR1 expression is correlated to higher WHO grade in astrocytomas, poor prognosis and invasion [57, 58]. FGFR1 is the most studied FGFR in GBM. In this study, we observed heterogeneous FGFR protein expression across cell lines, with FGFR2 absent in all tested GSC, consistent with Ohashi et al. findings, who reported reduced FGFR2 expression in high-grade gliomas [41]. FGFR1 protein appears to be the most highly expressed, with GC1 and GC3 showing higher levels than GC2 and GC4. TTFields specifically increased FGFR1 protein, suggesting its key role in GSC response to TTFields. To further investigate the role of FGFR1, we conducted downregulation experiments using siRNA targeting FGFR1. Similar to the effect of Pem, we observed a decrease in GSC survival following TTFields when FGFR1 expression was downregulated (data not shown), suggesting FGFR1 involvement in this response.

FGFR somatic mutations are among the most frequent molecular alterations occuring in GBM, leading to tumour growth [31, 59]. Of note, FGFR-TACC fusions, found in 3% of GBM, are oncogenic and clinical data have shown promising effects of FGFR inhibitors in GBM patients harbouring FGFR-TACC fusions [31, 44, 60, 61]. Several FGFR-targeting drugs are under clinical investigation [56]. Among them, we selected to investigate Pem, already used in clinics for locally advanced or metastatic cholangiosarcoma treatment. We demonstrate that Pem, combined with TTFields significantly reduces GSC survival. Pem is of particular interest from a clinical perspective since its intracranial activity has been demonstrated, meaning that this drug is able to cross the Blood Brain Barrier [62]. Moreover, Pem is currently under investigation in an open-label, phase II, monotherapy study (NCT05267106 – FIGHT 209) for recurrent GBM or other primary CNS tumours with an activating FGFR1-3 mutation or fusion/rearrangement [63]. Interestingly, a multicenter Phase II study evaluating the effect of infigratinib another selective FGFR1-3 inhibitor, recently demonstrates over a year of disease control in patients with tumours harbouring FGFR1/3 point mutations or FGFR3-TACC3 fusions [64]. In our study, Pem sensitized all GSC primocultures to TTFields. We investigated the mutational status of the cells used in this article and found that they do not present any fusions of FGFR (data not shown) meaning that Pem and TTFields are efficient regardless the presence of FGFR fusions. Moreover, MGMT promoter methylation is a key factor in GBM chemotherapy resistance. In this study, we used both MGMT un-methylated (GC1 and GC2) and MGMT-methylated cells (GC3 and GC4) and we observed that both TTFields and Pem treatment (alone or in combination) reduced survival across all cell lines. This confirms previous observations indicating that TTFields are efficient in GBM regardless of the MGMT methylation status [10, 65] and indicates that Pem could also benefit MGMT un-methylated GBM patients, known to be more resistant to TMZ. These results argue in favour that the therapeutic combination TTFields+Pem could be beneficial for all patients, even the less responder ones (MGMT un-methylated and without FGFR fusions).

An international phase III randomized trial (EF-32 - TRIDENT - NCT04471844) is ongoing to compare the efficacy of standard IR plus TMZ with the triple combination of IR, TMZ and concomitant TTFields in newly diagnosed GBM [27]. Here we demonstrated that Pem sensitizes GSC to the concomitant application of TTFields and IR by decreasing GSC survival and clonogenicity. TTFields and IR share common targets, the main one is DNA. In GBM, MPM and Non-Small Cell Lung Cancer (NSCLC) TTFields alter DNA repair through downregulation of BRCA1 (BReast CAncer gene 1) pathway genes and alteration of homologous recombination (HR) causing chromatid aberrations, DNA fragmentation and mitotic catastrophe [10, 15, 66]. In our study, we also demonstrate a delay in DNA damage repair, illustrated by increased γH2AX foci, when IR was followed by TTFields (IR>TTFields) as well as when TTFields were followed by IR (TTFields>IR). Noteworthy, in GC3 cells, combining TTFields with Pem further delayed the repair of DNA damages caused by IR, leading to cell death. This may result from impaired homology-mediated DNA repair, as FGFR inhibition, like TTFields, has been shown to attenuated the homology-mediated DNA-repair by altering the recruitment of Rad51 on DNA damages in GIST (GastroIntestinal Stromal Tumour) [67]. Similarly, it has been described that FGF signalling accelerates the kinetics of HR-mediated DNA damage repair in ovarian cancer [68]. Although the delay in DNA damage repair observed in GC3 when TTFields, Pem, and IR are combined is not seen in GC4, a decrease in GSC survival is still noted with this therapeutic combination in GC4. This means that another mechanism leading to GSC death, at least in GC4, might be involved. Oxidative stress regulation is crucial in anticancer therapy responses. ROS are unstable oxygen derivatives that have been extensively studied in various cancers. While essential for tumour function, excessive ROS can damage cancer cells [69]. IR induce ROS in cancer cells leading to DNA damage and cell death [25]. Similarly, the production of ROS is increased by TTFields, activating caspase signalling and apoptosis [70, 71]. Pem also raises intracellular ROS and oxidative stress [72]. Consequently, exploring whether Pem, IR and TTFields synergistically enhance ROS production and whether inhibiting ROS production or using antioxidant treatments can protect GSC from this combined therapy (IR+TTFields+Pem) warrants further investigation.

Moreover, articles in NSCLC and GBM differentiated cells showed that TTFields alone induce replicative stress by decreasing the expression of protein from the DNA replication complex genes MCM6, MCM10 (Mini-Chromosome Maintenance proteins) and FANC, leading to the instauration of a vulnerability environment sensitizing cells to IR and to the formation of Double-Strand Breaks (DSB) [15, 16, 24]. Pem also induces DNA damage in cancer cells [72]. Here, we did not observe an increase in DNA damage over time either with TTFields or Pem (alone or in combination) (data not shown). This could be explained by the fact that GSC are slow-cycling, which make them potentially more resistant to replicative stress. Moreover, they also have an increased DNA repair capacity compared to differentiated GBM cells [30]. Nevertheless, we observed the induction of GSC death when Pem was given concomitantly with TTFields (without IR), meaning that another mechanism not directly including DNA damage was involved. This might be apoptosis. Indeed, Kim et al. showed in GBM cells that the percentage of apoptotic cells was increased after TTFields [24] and Pace et al., shown in cell lines from different cancer types that Pem treatment leads to apoptosis [72]. Thus, it might be interesting to investigate the induction of apoptosis in our cells when TTFields and Pem are applicated concomitantly.

Altogether, this article highlights FGFR signalling as a key player in GSC resistance to TTFields and underlines its high potential as a therapeutic target in cancer, particularly in GBM. Thus, inhibition of FGFR by Pem administration concomitantly with TTFields might be a therapeutic combination highly relevant for improving the treatment of patients with GBM.

## Materials and methods

### Human tumour tissue collection, GSC isolation and culture

Fresh surgical tissue from newly diagnosed GBM patients was collected from the Neurosurgery department at Toulouse University Hospital as a part of the STEMRI clinical trial (NCT018872221) with written informed consent from each patient. Samples were processed in accordance with the Institution’s Human research Ethics Committee. For this study, tissue was isolated from different patients, which were histologically diagnosed as grade-IV astrocytoma according to the WHO criteria. In order to establish 4 primary GSC cell lines (GC1-4), samples were processed as described previously [73, 74]. The GSC were maintained in culture as 3D neurospheres in DMEM-F12 (GIBCO, New York, NY, USA) supplemented with B27 and N2 (Life Technologies, Carlsbad, CA, USA) and EGF and FGF2 (Peprotech, Rocky Hill, NJ, USA) in a CO_2_ incubator (5%) at 37 °C. Cells were used between the 2nd and 12th passage in order to avoid any stem cell property loss and tested for mycoplasma contamination.

### Tumour treating fields (TTFields) treatment

Neurospheres were dissociated and cells were seeded in 2ml of culture medium into Inovitro^TM^ ceramic dishes (Novocure, Haifa, Israel). Dishes were placed onto a base plate connected to a generator administrating TTFields at a frequency of 200 kHz and an intensity of 1.7 V/cm for 72 h (Novocure, Haifa, Israel).

### Cells irradiation

Cells were dissociated and maintained in complete medium for 18 h. Cells were then irradiated with an irradiator XRAD SmART+ (Precision X-ray Inc., Madison, WI, USA). For experiments with IR combined with TTFields, cells were submitted to TTFields within 1 h after the end of IR treatment.

### Pemigatinib treatment

Pemigatinib (TargetMol Chemicals, Wellesley Hills, MA, USA) was diluted firstly in DMSO (Sigma-Aldrich, Saint-Quentin Fallavier, France) and then used at a final concentration of 125nM diluted in complete medium. DMSO was used as control condition. Pem was added in ceramic dishes at cell seeding and maintained all over TTFields treatment.

### Cell growth analysis by cell counting

After treatments (TTFields, IR and/or Pem) cells were harvested, centrifuged and resuspended in 200 µl of complete medium. Total number of live cells per condition was determined using an automated cell counter (Countess II FL, Life Technologies, Carlsbad, CA, USA).

### Clonogenic assay

After TTFields application (w/o Pem or DMSO), cells were dissociated and 500cells/well were plated in complete medium in 96-wells plates (Corning, New York, NY, USA) and exposed to IR and/or Pem or DMSO. After 7days of incubation at 37 °C and 5% CO_2_, whole wells were imaged using an Operetta CLS Imaging system (Perkin Elmer, Waltham, MA, USA) and the number of sphere per well was quantified.

### Quantitative real-time PCR

Total RNA was isolated using RNeasy® Plus Micro kit (Qiagen, Venlo, The Netherlands) and then reverse-transcribed using Prime Script RT Reagent kit (TAKARA, Kusatsu, Japan). Real-time qPCR reactions were carried out using SsoFast^TM^ EvaGreen® Supermix dye (Biorad, Marnes-la-Coquette, France) and the ABI-StepOnePlus Detection System (Applied Biosystems, Waltham, MA, USA). GAPDH was used as endogenous control in the ΔCt analysis. The different primers used in this study were described in Supplementary Table 6. GAPDH and FGFR1 primers were purchased from Invitrogen (Waltham, MA, USA) and FGFR2-4 primers were purchased from Eurogentech (Liege, Belgium).

### Cytotoxicity evaluation by LIVE/DEAD^TM^ staining

Dissociated GSC were seeded into Inovitro^TM^ dishes in 2 ml of complete medium supplemented with Pem or DMSO as control. After 72 h of TTFields application, cells were harvested, washed in PBS (Sigma-Aldrich, Saint-Quentin Fallavier, France) and stained using LIVE/DEAD^TM^ viability/cytotoxicity kit (Invitrogen, Carlsbad, CA, USA) for 30 min in the dark at room temperature. This kit is based on a double staining with calcein AM to discriminate live cells and ethidium homodimer to identify dead cells. The percentage of live and dead cells was quantified by flow cytometry using a MACSQuant Analyzer 10 cytometer (Miltenyi Biotec, Bergisch Gladbach, Germany). For experiments with IR, cells were irradiated prior TTFields treatment and TTFields treatment was started within 1 h after IR.

### Western blotting

Cells were lysed in RIPA buffer (Sigma-Aldrich, Saint-Quentin Fallavier, France) complemented with proteases and phosphatases inhibitors (Sigma-Aldrich, Saint-Quentin Fallavier, France). Quantity of proteins in each sample was assessed using Bradford Reagent (Biorad, Marnes-la-Coquette, France) and 30 µg of proteins were separated on a 10% SDS-PAGE, electroblotted onto PVDF membranes (Amersham^TM^) (Thermo Fisher Scientific, Waltham, MA, USA). Membranes were then blocked into 10% milk for 1 h. Primary antibody against Phosphorylated FRS2 (Tyr196) (#3864, Cell Signalling, Danvers, MA, USA), FGFR1 (#9740, Cell Signalling, Danvers, MA, USA), FGFR2 (#A23298, ABclonal, Woburn, MA, USA), FGFR3 (#A0404, ABclonal, Woburn, MA, USA), FGFR4 (#A9197, ABclonal, Woburn, MA, USA) and against GAPDH (#CB1001, Millipore, Molsheim, France) were incubated overnight. Membranes were then incubated with HRP-linked secondary antibodies (anti-mouse or anti-rabbit, Abcam Cambridge, United Kingdom) and the reaction was developed with Western ECL substrate (Thermo Fisher Scientific, Waltham, MA, USA). Signal was revealed with a Chemidoc (Biorad, Hercules, CA, USA) and data were analysed with ImageLab Software (Version 6.1) (Biorad, Hercules, CA, USA).

### Flow cytometry (*γH2AX analysis)*

After 72 h of TTFields application w/o DMSO or Pem, cells were harvested, washed in PBS (Sigma-Aldrich, Saint-Quentin Fallavier, France) and fixed for 1 h at −20 °C in ice-cold 70% ethanol (VWR, Radnor, PA, USA). Cells were then washed with Cell staining Buffer (Biolegend, San Diego, CA, USA) and incubated for 1 h at 4 °C in the dark with primary Anti-H2A.X-Phosphorylated (Ser139) Antibody conjugated to Alexa Fluor® 488 (#613405, BioLegend, San Diego, CA, USA) or Alexa Fluor® 488 Mouse IgG1, κ Isotype Ctrl Antibody (#400133, BioLegend, San Diego, CA, USA).

After a wash in PBS (Sigma-Aldrich, Saint-Quentin Fallavier, France), fluorescent signal was measured using a MACSQuant VYB cytometer (Miltenyi Biotec, Bergisch Gladbach, Germany). For each sample, a total of at least 10,000 events was recorded and data were analysed using FlowJo^TM^ v10.9 Software (BD Life Sciences, Franklin Lakes, NJ, USA). To evaluate the marker expression, Specific Fluorescence Index (SFI) was determined using the Geometric mean fluorescence intensity (Geomean). The SFI was calculated as previously described with the formula SFI = (Geomean antibody – Geomean isotype control)/Geomean isotype control [73, 75].

### Statistical analysis

Results are represented as means ± SEM of at least 3 independent experiments. Each dot on graphs represent one independent experiment. The number of samples was chosen to maximize statistical robustness while respecting biological and experimental constraints. Significant differences were calculated using Student’s unpaired t-test for evaluating the effect of monotherapy (TTFields or IR alone) and one-way ANOVA followed by Tukey’s or Sidak’s multiple comparison test for analyzing the impact of multiple therapeutic combinations (IR and/or TTFields and/or Pem). All statistical analyses were performed using GraphPad Prism version 10.1.2 for Windows (GraphPad Software, Boston, MA, USA). The significant threshold (α) was set at 0.05. For clarity, not all statistical test results are displayed in the graphs, full summary statistics are provided in Supplementary Tables 7–10.

BLISS analysis was performed to determine if the interactions between the treatments (Pem, TTFields and IR) are synergistic, additive or antagonist as described previously [47, 76]. Briefly, for two agents, the expected total response to the combination treatment was calculated as Fractional response to treatment A (*F*_*a*_) + Fractional response to treatment B (*F*_*b*_) – *F*_*a*_ × *F*_*b*_. For three agents, the expected total response was calculated as *F*_*a*_ + *F*_*b*_ + *F*_*c*_ – *F*_*a*_ × *F*_*b*_ – *F*_*a*_ × *F*_*c*_ – *F*_*b*_ × *F*_*c*_ + *F*_*a*_ × *F*_*b*_ × *F*_*c*_. Additivity was assumed if the ratio of the actual total response to the expected total response ranged for 0.9 to 1.1. If the was less than 0.9, the effect was considered antagonistic, while if the ratio exceeded 1.1 it was considered synergistic.

## Supplementary information

Supplementary Figures

Supplementary Table 1

Supplementary Table 2

Supplementary Table 3

Supplementary Table 4

Supplementary Table 5

Supplementary Table 6

Supplementary Table 7

Supplementary Table 8

Supplementary Table 9

Supplementary Table 10

Supplementary Methods

Original western blots

## Data Availability

All data generated or analysed during this study are included in this published article.
