# Supplementary material for: FGFR inhibition as a new therapeutic strategy to sensitize glioblastoma stem cells to tumor treating fields
Source: Cell Death Discov. 2025 Jun 4;11:265. doi: 10.1038/s41420-025-02542-5 (PMC12137614; doi:10.1038/s41420-025-02542-5)
Supplement: Supplementary file 1 — Supplementary Figures [file 41420_2025_2542_MOESM1_ESM.pdf]

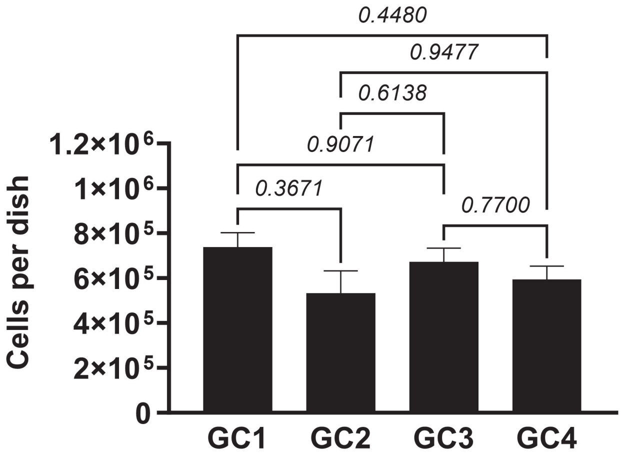

Supplementary Figure 1 : Proliferation capacity of the 4 GSC primocultures.

Graph represents the mean +/- SEM of the number of live cells determined by cell count in each dish after 72hours of culture at 5%CO2, 37°C. Errors bars show means+/-SEM of N≥3 experiments.

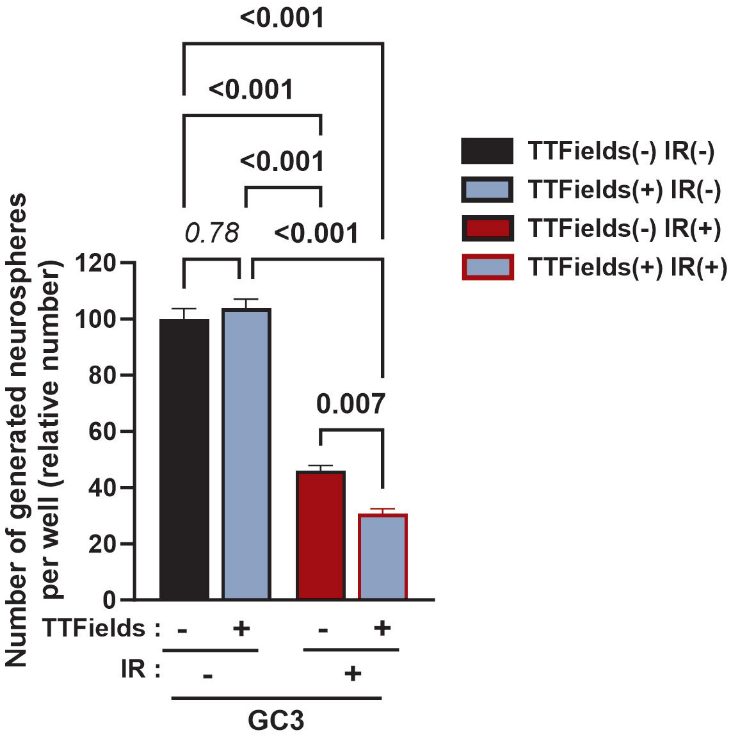

<u>Supplementary Figure 2 :</u> Clonogenic assay with GC3 treated by IR followed by TTFields. Dissociated GSC were irradiated at 4Gy (IR(+)) or not-irradiated as control (IR(-)) and within 1hour after IR treatment, cells were seeded into Inovitro® dishes and submitted to TTFields for 72hours (TTFields(+)) or untreated (TTFields(-)). Cells were then dissociated and incubated for 7days in stem cell medium at 37°C. The number of neurospheres was then counted in each well. Graph represents the mean+/-SEM of the percentage of formed neurospheres per well (normalized to non-irradiated, TTFields untreated condition (TTFields(-), IR(-)). Errors bars show means+/-SEM of N=5 wells per condition.

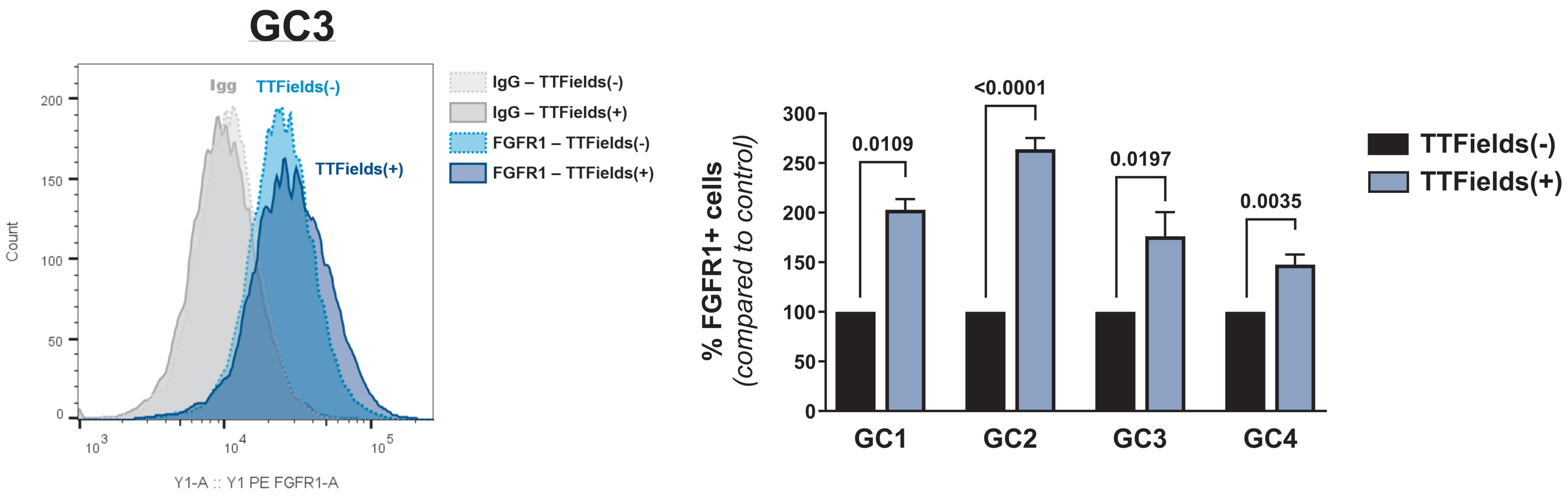

<u>Supplementary Figure 3 :</u> Flow cytometry analysis of FGFR1 protein expression.
Graph represents means+/-SEM of the percentage of FGFR1 positive cells of all viable cells in TTFields-treated GSC (TTFields(+)) normalized to untreated GSC (TTFields(-)). Errors bars show means+/-SEM of N≥3 experiments.

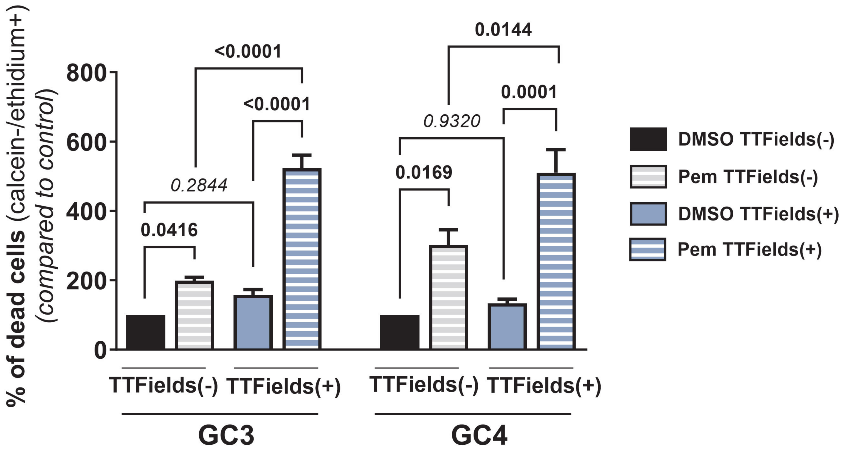

<u>Supplementary Figure 4:</u> Pem treatment combined with TTFields induces GSC death. Percentage of dead cells determined by flow cytometry. Graph represents means +/- SEM of the percentage of cells negative for calcein (calcein-) and positive for ethidium staining (ethidium+) in GC3 and GC4 treated with TTFields for 72hours (TTFields(+)) or untreated (TTFields(-)) combined with treatment with Pem (Pem) or DMSO as control (DMSO). Results are normalized to TTFields(-) condition treated with DMSO for each cell line. Errors bars show means+/-SEM of N≥3 experiments.

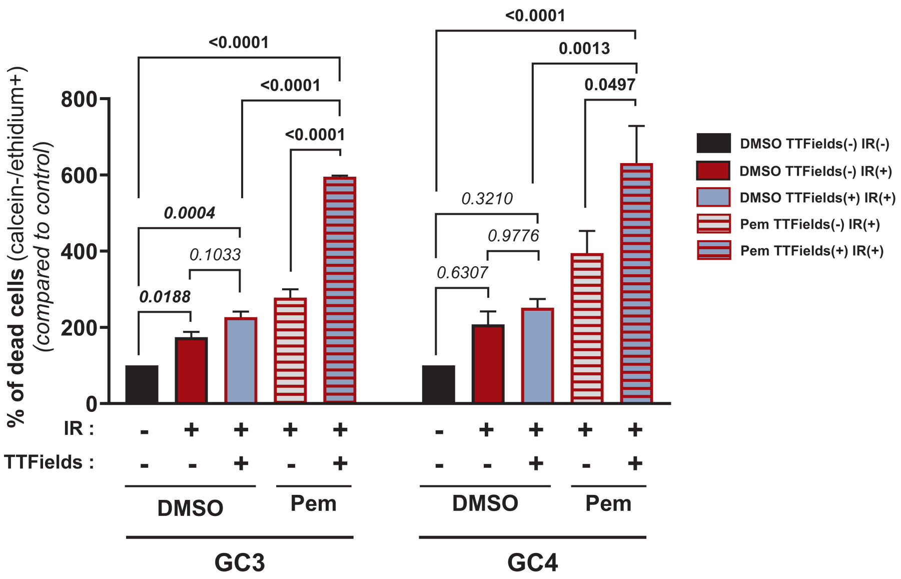

<u>Supplementary Figure 5 :</u> Pem treatment increases GSC death in response to the concomitant application of TTFields and IR.

Percentage of dead cells determined by flow cytometry. Graph represents means +/- SEM of the percentage of cells negative for calcein (calcein-) and positive for ethidium staining (ethidium+) in GC3 and GC4 irradiated (IR(+)) or non-irradiated (IR(-)) and then treated with TTFields for 72hours (TTFields(+)) or not (TTFields(-)) combined with a treatment with Pem (Pem) or DMSO as control (DMSO). Results are normalized to non-irradiated, TTFields-untreated condition treated with DMSO for each cell line. Errors bars show means+/-SEM of N≥3 experiments.

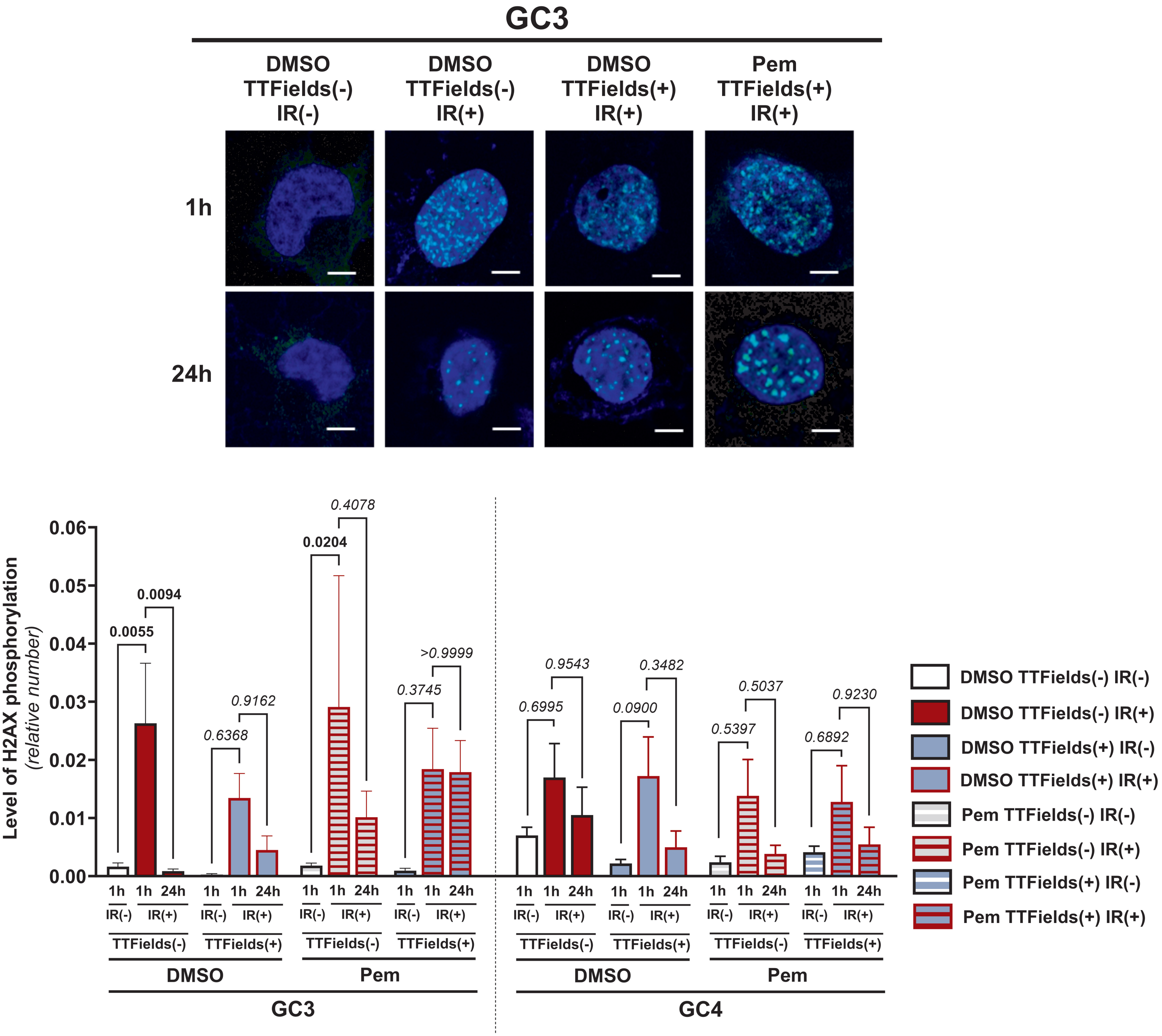

<u>Supplementary Figure 6 :</u> Influence of treatment with Pem, TTFields and IR on H2AX phosphorylation levels in GC3 and GC4.

Immunocytochemistry of phosphorylated (Ser139) H2AX (γH2AX) in GC3 and GC4 treated with TTFields (TTFields(+)) or un-treated (TTFields(-)) for 72hours in combination with treatment with Pem (Pem) or DMSO as control (DMSO) and followed by irradiation (IR(+)) or not (IR(-)). Cells were stained 1hour after IR (1h) or 24hours after IR (24h). Graph represents the mean+/-SEM of the relative amount of green staining (γH2AX) per nucleus (blue staining, DAPI) (see Supplementary Methods). Errors bars show means+/-SEM of N>10 nuclei quantified per condition. Scale bars 10µm.

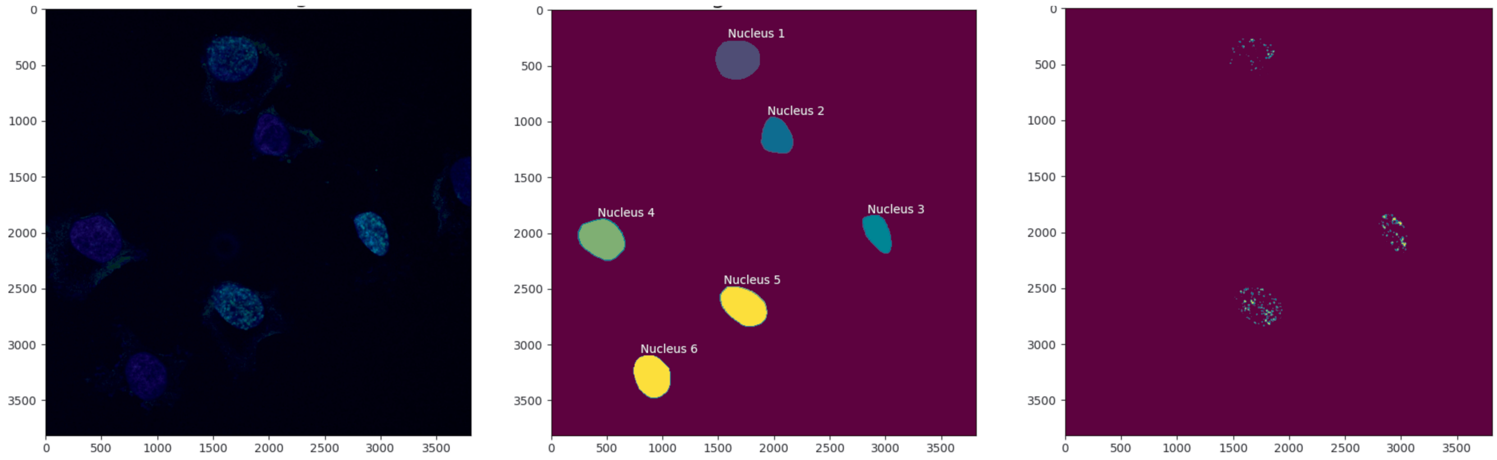

(A) Raw Image containing several nuclei.

(B) Result of the nuclei detection pipeline

(C) Pixels counted as "green"

Supplementary Figure 7 : Sample image from the γH2AX foci quantification pipeline.
