# Supplementary material for: FGFR inhibition as a new therapeutic strategy to sensitize glioblastoma stem cells to tumor treating fields
Source: Cell Death Discov. 2025 Jun 4;11:265. doi: 10.1038/s41420-025-02542-5 (PMC12137614; doi:10.1038/s41420-025-02542-5)
Supplement: Supplementary file 2 — Supplementary Table 1 [file 41420_2025_2542_MOESM2_ESM.docx]

| **Figure 1C** | **GC1** | **GC2** | **GC3** | **GC4** |
| --- | --- | --- | --- | --- |
| Bliss Score | 1.13 | 1.31 | 1.24 | 1.10 |
| Effect | **Synergy** | **Synergy** | **Synergy** | Additivity |

**Supplementary Table 1 :** Calculation of Bliss Index (Figure 1C)
