# Supplementary material for: FGFR inhibition as a new therapeutic strategy to sensitize glioblastoma stem cells to tumor treating fields
Source: Cell Death Discov. 2025 Jun 4;11:265. doi: 10.1038/s41420-025-02542-5 (PMC12137614; doi:10.1038/s41420-025-02542-5)
Supplement: Supplementary file 5 — Supplementary Table 4 [file 41420_2025_2542_MOESM5_ESM.docx]

| **Figure 4A** | **GC3** | **GC4** |
| --- | --- | --- |
| Bliss Score | 2.37 | 1.72 |
| Effect | **Synergy** | **Synergy** |

**Supplementary Table 4 :** Calculation of Bliss Index (Figure 4A)
