# Supplementary material for: FGFR inhibition as a new therapeutic strategy to sensitize glioblastoma stem cells to tumor treating fields
Source: Cell Death Discov. 2025 Jun 4;11:265. doi: 10.1038/s41420-025-02542-5 (PMC12137614; doi:10.1038/s41420-025-02542-5)
Supplement: Supplementary file 7 — Supplementary Table 6 [file 41420_2025_2542_MOESM7_ESM.docx]

| **GENE** | **PRIMER FORWARD (5’ – 3’)** | **PRIMER REVERSE (3’ – 5’)** |
| --- | --- | --- |
| GAPDH | TGCACCACCAACTGCTTAGC | GGCATGGACTGTGGTCATGAG |
| FGFR1 | CAGTCCATCCATGAACTCTG | GTCACACGGTTGGGTTTGTC |
| FGFR2 | GCCACAGAGAAGGACCTGTCTGATCTGGT | CTCCTCGGGGACACGGTTAATGTCATAGG |
| FGFR3 | CTGAAGAACGGCAGGGAGTT | CCAGGCTCCACTGCTGATG |
| FGFR4 | GAGGAGGACCCCACATGGA | GTACAGGATGATGTCCGTATACCT |

**Supplementary Table 6 :** Forward and reverse primer sequence used for qPCR detection.
