# Supplementary material for: FGFR inhibition as a new therapeutic strategy to sensitize glioblastoma stem cells to tumor treating fields
Source: Cell Death Discov. 2025 Jun 4;11:265. doi: 10.1038/s41420-025-02542-5 (PMC12137614; doi:10.1038/s41420-025-02542-5)
Supplement: Supplementary file 8 — Supplementary Table 7 [file 41420_2025_2542_MOESM8_ESM.docx]

|  |  | **Comparison** *(Unpaired t test – two-tailed)* | **P value** | **Significance** |
| --- | --- | --- | --- | --- |
| **Figure 1A** | **GC1** | TTFields(-) *vs.* TTFields(+) | <0,0001 | **** |
|  | **GC2** | TTFields(-) *vs.* TTFields(+) | <0.0001 | **** |
|  | **GC3** | TTFields(-) *vs.* TTFields(+) | <0.0001 | **** |
|  | **GC4** | TTFields(-) *vs.* TTFields(+) | <0.0001 | **** |
|  |  | **Comparison** *(Unpaired t test – two-tailed)* | **P value** | **Significance** |
| **Figure 1B** | **GC1** | TTFields(-) *vs.* TTFields(+) | 0.0412 | * |
|  | **GC2** | TTFields(-) *vs.* TTFields(+) | 0.0035 | ** |
|  | **GC3** | TTFields(-) *vs.* TTFields(+) | 0.0177 | * |
|  | **GC4** | TTFields(-) *vs.* TTFields(+) | 0.0140 | * |
|  |  | **Comparison**  *(One-way ANOVA + Tukey’s multiple comparisons test)* | **Adjusted P value** | **Significance** |
| **Figure 1C** | **GC1** | TTFields(-) IR(-) *vs.* TTFields(+) IR(-) | 0.0112 | * |
|  |  | TTFields(-) IR(-) *vs.* TTFields(-) IR(+) | <0.0001 | **** |
|  |  | TTFields(-) IR(-) *vs.* TTFields(+) IR(+) | <0.0001 | **** |
|  |  | TTFields(+) IR(-) *vs.* TTFields(-) IR(+) | <0.0001 | **** |
|  |  | TTFields(+) IR(-) *vs.* TTFields(+) IR(+) | <0.0001 | **** |
|  |  | TTFields(-) IR(+) *vs.* TTFields(+) IR(+) | 0.0131 | * |
|  | **GC2** | TTFields(-) IR(-) *vs.* TTFields(+) IR(-) | 0.0954 | ns |
|  |  | TTFields(-) IR(-) *vs.* TTFields(-) IR(+) | 0.0004 | *** |
|  |  | TTFields(-) IR(-) *vs.* TTFields(+) IR(+) | <0.0001 | **** |
|  |  | TTFields(+) IR(-) *vs.* TTFields(-) IR(+) | 0.0308 | * |
|  |  | TTFields(+) IR(-) *vs.* TTFields(+) IR(+) | <0.0001 | **** |
|  |  | TTFields(-) IR(+) *vs.* TTFields(+) IR(+) | 0.0302 | * |
|  | **GC3** | TTFields(-) IR(-) *vs.* TTFields(+) IR(-) | 0.1502 | ns |
|  |  | TTFields(-) IR(-) *vs.* TTFields(-) IR(+) | 0.0004 | *** |
|  |  | TTFields(-) IR(-) *vs.* TTFields(+) IR(+) | <0.0001 | **** |
|  |  | TTFields(+) IR(-) *vs.* TTFields(-) IR(+) | 0.0184 | * |
|  |  | TTFields(+) IR(-) *vs.* TTFields(+) IR(+) | <0.0001 | **** |
|  |  | TTFields(-) IR(+) *vs.* TTFields(+) IR(+) | 0.0133 | * |
|  | **GC4** | TTFields(-) IR(-) *vs.* TTFields(+) IR(-) | 0.7103 | ns |
|  |  | TTFields(-) IR(-) *vs.* TTFields(-) IR(+) | <0.0001 | **** |
|  |  | TTFields(-) IR(-) *vs.* TTFields(+) IR(+) | <0.0001 | **** |
|  |  | TTFields(+) IR(-) *vs.* TTFields(-) IR(+) | <0.0001 | **** |
|  |  | TTFields(+) IR(-) *vs.* TTFields(+) IR(+) | <0.0001 | **** |
|  |  | TTFields(-) IR(+) *vs.* TTFields(+) IR(+) | 0.2438 | ns |

**Supplementary Table 7 :** Summary statistics of data presented in Figure 1. *(*p<0.05 ; **p<0.01 ; ***p<0.001 ; ****p<0.0001 ; ns : not-significant).*
