# Supplementary material for: FGFR inhibition as a new therapeutic strategy to sensitize glioblastoma stem cells to tumor treating fields
Source: Cell Death Discov. 2025 Jun 4;11:265. doi: 10.1038/s41420-025-02542-5 (PMC12137614; doi:10.1038/s41420-025-02542-5)
Supplement: Supplementary file 9 — Supplementary Table 8 [file 41420_2025_2542_MOESM9_ESM.docx]

|  |  | **Comparison**  *(One-way ANOVA + Dunnett’s multiple comparisons test)* | | **P value** | **Significance** |
| --- | --- | --- | --- | --- | --- |
| **Figure 2A** | **GC1** | FGFR1 *vs.* FGFR2 | | <0.0001 | **** |
|  |  | FGFR1 *vs.* FGFR3 | | <0.0001 | **** |
|  |  | FGFR1 *vs.* FGFR4 | | <0.0001 | **** |
|  | **GC2** | FGFR1 *vs.* FGFR2 | | <0.0001 | **** |
|  |  | FGFR1 *vs.* FGFR3 | | <0.0001 | **** |
|  |  | FGFR1 *vs.* FGFR4 | | <0.0001 | **** |
|  | **GC3** | FGFR1 *vs.* FGFR2 | | 0.0009 | *** |
|  |  | FGFR1 *vs.* FGFR3 | | 0.0009 | *** |
|  |  | FGFR1 *vs.* FGFR4 | | 0.0009 | *** |
|  | **GC4** | FGFR1 *vs.* FGFR2 | | 0.0001 | *** |
|  |  | FGFR1 *vs.* FGFR3 | | 0.0001 | *** |
|  |  | FGFR1 *vs.* FGFR4 | | 0.0001 | *** |
|  |  | **Comparison** *(Unpaired t test – two-tailed)* | | **Adjusted P value** | **Significance** |
| **Figure 2C** | **FGFR1** | GC3 | TTFields(-) *vs.* TTFields(+) | 0.0205 | * |
|  |  | GC4 | TTFields(-) *vs.* TTFields(+) | 0.0695 | ns |
|  | **FGFR2** | GC3 | TTFields(-) *vs.* TTFields(+) | 0.0219 | * |
|  |  | GC4 | TTFields(-) *vs.* TTFields(+) | 0.0092 | ** |
|  | **FGFR3** | GC3 | TTFields(-) *vs.* TTFields(+) | 0.0201 | * |
|  |  | GC4 | TTFields(-) *vs.* TTFields(+) | 0.1879 | ns |
|  | **FGFR4** | GC3 | TTFields(-) *vs.* TTFields(+) | 0.0050 | ** |
|  |  | GC4 | TTFields(-) *vs.* TTFields(+) | 0.0017 | ** |

**Supplementary Table 8 :** Summary statistics of data presented in Figure 2. *(*p<0.05 ; **p<0.01 ; ***p<0.001 ; ****p<0.0001 ; ns : not-significant).*
