# Supplementary material for: FGFR inhibition as a new therapeutic strategy to sensitize glioblastoma stem cells to tumor treating fields
Source: Cell Death Discov. 2025 Jun 4;11:265. doi: 10.1038/s41420-025-02542-5 (PMC12137614; doi:10.1038/s41420-025-02542-5)
Supplement: Supplementary file 10 — Supplementary Table 9 [file 41420_2025_2542_MOESM10_ESM.docx]

|  |  | **Comparison**  *(One-way ANOVA + Tukey’s multiple comparisons test)* | **Adjusted P value** | **Significance** |
| --- | --- | --- | --- | --- |
| **Figure 3B** | **GC1** | DMSO TTFields(-) *vs.* Pem TTFields(-) | <0.0001 | **** |
|  |  | DMSO TTFields(-) *vs.* DMSO TTFields(+) | 0.0002 | *** |
|  |  | DMSO TTFields(-) *vs.* Pem TTFields(+) | <0.0001 | **** |
|  |  | Pem TTFields(-) *vs.* DMSO TTFields(+) | <0.0001 | **** |
|  |  | Pem TTFields(-) *vs.* Pem TTFields(+) | 0.0425 | * |
|  |  | DMSO TTFields(+) *vs.* Pem TTFields(+) | <0.0001 | **** |
|  | **GC2** | DMSO TTFields(-) *vs.* Pem TTFields(-) | <0.0001 | **** |
|  |  | DMSO TTFields(-) *vs.* DMSO TTFields(+) | 0.0344 | * |
|  |  | DMSO TTFields(-) *vs.* Pem TTFields(+) | <0.0001 | **** |
|  |  | Pem TTFields(-) *vs.* DMSO TTFields(+) | <0.0001 | **** |
|  |  | Pem TTFields(-) *vs.* Pem TTFields(+) | 0.0251 | * |
|  |  | DMSO TTFields(+) *vs.* Pem TTFields(+) | <0.0001 | **** |
|  | **GC3** | DMSO TTFields(-) *vs.* Pem TTFields(-) | <0.0001 | **** |
|  |  | DMSO TTFields(-) *vs.* DMSO TTFields(+) | 0.0127 | * |
|  |  | DMSO TTFields(-) *vs.* Pem TTFields(+) | <0.0001 | **** |
|  |  | Pem TTFields(-) *vs.* DMSO TTFields(+) | 0.0174 | * |
|  |  | Pem TTFields(-) *vs.* Pem TTFields(+) | 0.0029 | ** |
|  |  | DMSO TTFields(+) *vs.* Pem TTFields(+) | <0.0001 | **** |
|  | **GC4** | DMSO TTFields(-) *vs.* Pem TTFields(-) | <0.0001 | **** |
|  |  | DMSO TTFields(-) *vs.* DMSO TTFields(+) | <0.0001 | **** |
|  |  | DMSO TTFields(-) *vs.* Pem TTFields(+) | <0.0001 | **** |
|  |  | Pem TTFields(-) *vs.* DMSO TTFields(+) | 0.2316 | ns |
|  |  | Pem TTFields(-) *vs.* Pem TTFields(+) | 0.0001 | *** |
|  |  | DMSO TTFields(+) *vs.* Pem TTFields(+) | <0.0001 | **** |
|  |  | **Comparison**  *(One-way ANOVA + Tukey’s multiple comparisons test)* | **Adjusted P value** | **Significance** |
| **Figure 3C** | **GC3** | DMSO TTFields(-) *vs.* Pem TTFields(-) | 0.4509 | ns |
|  |  | DMSO TTFields(-) *vs.* DMSO TTFields(+) | 0.7813 | ns |
|  |  | DMSO TTFields(-) *vs.* Pem TTFields(+) | 0.0006 | *** |
|  |  | Pem TTFields(-) *vs.* DMSO TTFields(+) | 0.9261 | ns |
|  |  | Pem TTFields(-) *vs.* Pem TTFields(+) | 0.0034 | ** |
|  |  | DMSO TTFields(+) *vs.* Pem TTFields(+) | 0.0017 | ** |
|  | **GC4** | DMSO TTFields(-) *vs.* Pem TTFields(-) | 0.0188 | * |
|  |  | DMSO TTFields(-) *vs.* DMSO TTFields(+) | 0.8799 | ns |
|  |  | DMSO TTFields(-) *vs.* Pem TTFields(+) | <0.0001 | **** |
|  |  | Pem TTFields(-) *vs.* DMSO TTFields(+) | 0.0678 | ns |
|  |  | Pem TTFields(-) *vs.* Pem TTFields(+) | 0.0076 | ** |
|  |  | DMSO TTFields(+) *vs.* Pem TTFields(+) | <0.0001 | **** |
|  |  | **Comparison**  *(One-way ANOVA + Tukey’s multiple comparisons test)* | **Adjusted P value** | **Significance** |
| **Figure 3D** | **GC3** | DMSO TTFields(-) *vs.* Pem TTFields(-) | 0.1266 | ns |
|  |  | DMSO TTFields(-) *vs.* DMSO TTFields(+) | 0.4976 | ns |
|  |  | DMSO TTFields(-) *vs.* Pem TTFields(+) | <0.0001 | **** |
|  |  | Pem TTFields(-) *vs.* DMSO TTFields(+) | 0.9620 | ns |
|  |  | Pem TTFields(-) *vs.* Pem TTFields(+) | 0.0038 | ** |
|  |  | DMSO TTFields(+) *vs.* Pem TTFields(+) | 0.0002 | *** |
|  | **GC4** | DMSO TTFields(-) *vs.* Pem TTFields(-) | 0.0004 | *** |
|  |  | DMSO TTFields(-) *vs.* DMSO TTFields(+) | 0.0204 | * |
|  |  | DMSO TTFields(-) *vs.* Pem TTFields(+) | <0.0001 | **** |
|  |  | Pem TTFields(-) *vs.* DMSO TTFields(+) | 0.6992 | ns |
|  |  | Pem TTFields(-) *vs.* Pem TTFields(+) | 0.0103 | ** |
|  |  | DMSO TTFields(+) *vs.* Pem TTFields(+) | 0.0002 | *** |

**Supplementary Table 9 :** Summary statistics of data presented in Figure 3. *(*p<0.05 ; **p<0.01 ; ***p<0.001 ; ****p<0.0001 ; ns : not-significant).*
