# Supplementary material for: FGFR inhibition as a new therapeutic strategy to sensitize glioblastoma stem cells to tumor treating fields
Source: Cell Death Discov. 2025 Jun 4;11:265. doi: 10.1038/s41420-025-02542-5 (PMC12137614; doi:10.1038/s41420-025-02542-5)
Supplement: Supplementary file 11 — Supplementary Table 10 [file 41420_2025_2542_MOESM11_ESM.docx]

|  |  | **Comparison**  *(One-way ANOVA + Tukey’s multiple comparisons test)* | | **Adjusted P value** | **Significance** |
| --- | --- | --- | --- | --- | --- |
| **Figure 4A** | **GC3** | DMSO TTFields(-) IR(-) *vs.* DMSO TTFields(-) IR(+) | | 0.5015 | ns |
|  |  | DMSO TTFields(-) IR(-) *vs.* DMSO TTFields(+) IR(+) | | 0.0850 | ns |
|  |  | DMSO TTFields(-) IR(-) *vs.* Pem TTFields(-) IR(+) | | 0.0124 | * |
|  |  | DMSO TTFields(-) IR(-) *vs.* Pem TTFields(+) IR(+) | | <0.0001 | **** |
|  |  | DMSO TTFields(-) IR(+) *vs.* DMSO TTFields(+) IR(+) | | 0.6987 | ns |
|  |  | DMSO TTFields(-) IR(+) *vs.* Pem TTFields(-) IR(+) | | 0.1551 | ns |
|  |  | DMSO TTFields(-) IR(+) *vs.* Pem TTFields(+) IR(+) | | <0.0001 | **** |
|  |  | DMSO TTFields(+) IR(+) *vs.* Pem TTFields(-) IR(+) | | 0.7244 | ns |
|  |  | DMSO TTFields(+) IR(+) *vs.* Pem TTFields(+) IR(+) | | <0.0001 | **** |
|  |  | Pem TTFields(-) IR(+) *vs.* Pem TTFields(+) IR(+) | | 0.0002 | *** |
|  | **GC4** | DMSO TTFields(-) IR(-) *vs.* DMSO TTFields(-) IR(+) | | 0.3556 | ns |
|  |  | DMSO TTFields(-) IR(-) *vs.* DMSO TTFields(+) IR(+) | | 0.0589 | ns |
|  |  | DMSO TTFields(-) IR(-) *vs.* Pem TTFields(-) IR(+) | | 0.0006 | *** |
|  |  | DMSO TTFields(-) IR(-) *vs.* Pem TTFields(+) IR(+) | | <0.0001 | **** |
|  |  | DMSO TTFields(-) IR(+) *vs.* DMSO TTFields(+) IR(+) | | 0.8074 | ns |
|  |  | DMSO TTFields(-) IR(+) *vs.* Pem TTFields(-) IR(+) | | 0.0212 | * |
|  |  | DMSO TTFields(-) IR(+) *vs.* Pem TTFields(+) IR(+) | | <0.0001 | **** |
|  |  | DMSO TTFields(+) IR(+) *vs.* Pem TTFields(-) IR(+) | | 0.1563 | ns |
|  |  | DMSO TTFields(+) IR(+) *vs.* Pem TTFields(+) IR(+) | | 0.0001 | *** |
|  |  | Pem TTFields(-) IR(+) *vs.* Pem TTFields(+) IR(+) | | 0.0113 | * |
|  |  | **Comparison**  *(One-way ANOVA + Šídák's multiple comparisons test)* | | **Adjusted P value** | **Significance** |
| **Figure 4B** | **GC3** | 1h  IR(-) | DMSO TTFields(-) IR(-) *vs.* DMSO TTFields(+) IR(-) | 0.9985 | ns |
|  |  |  | DMSO TTFields(-) IR(-) *vs.* Pem TTFields(-) IR(-) | >0.9999 | ns |
|  |  |  | DMSO TTFields(-) IR(-) *vs.* Pem TTFields(+) IR(-) | 0.9862 | ns |
|  |  |  | DMSO TTFields(+) IR(-) *vs.* Pem TTFields(+) IR(-) | 0.9999 | ns |
|  |  |  | Pem TTFields(-) IR(-) *vs.* Pem TTFields(+) IR(-) | 0.9892 | ns |
|  |  | 1h IR(+) | DMSO TTFields(-) IR(+) *vs.* DMSO TTFields(+) IR(+) | 0.9951 | ns |
|  |  |  | DMSO TTFields(-) IR(+) *vs.* Pem TTFields(-) IR(+) | 0.9785 | ns |
|  |  |  | DMSO TTFields(-) IR(+) *vs.* Pem TTFields(+) IR(+) | >0.9999 | ns |
|  |  |  | DMSO TTFields(+) IR(+) *vs.* Pem TTFields(+) IR(+) | 0.9748 | ns |
|  |  |  | Pem TTFields(-) IR(+) *vs.* Pem TTFields(+) IR(+) | 0.9337 | ns |
|  |  | 24h IR(+) | DMSO TTFields(-) IR(+) *vs.* DMSO TTFields(+) IR(+) | 0.4281 | ns |
|  |  |  | DMSO TTFields(-) IR(+) *vs.* Pem TTFields(-) IR(+) | 0.9982 | ns |
|  |  |  | DMSO TTFields(-) IR(+) *vs.* Pem TTFields(+) IR(+) | 0.0041 | ** |
|  |  |  | DMSO TTFields(+) IR(+) *vs.* Pem TTFields(+) IR(+) | 0.0478 | * |
|  |  |  | Pem TTFields(-) IR(+) *vs.* Pem TTFields(+) IR(+) | 0.0026 | ** |

|  | **Comparison**  *(One-way ANOVA* + Šídák's multiple comparisons test) | | | **Adjusted P value** | | **Significance** | |
| --- | --- | --- | --- | --- | --- | --- | --- |
| **Figure 4B** | **GC4** | 1h IR(-) | DMSO TTFields(-) IR(-) *vs.* DMSO TTFields(+) IR(-) | 0.9947 | | ns | |
|  |  |  | DMSO TTFields(-) IR(-) *vs.* Pem TTFields(-) IR(-) | | 0.9999 | ns | |
|  |  |  | DMSO TTFields(-) IR(-) *vs.* Pem TTFields(+) IR(-) | | >0.9999 | ns | |
|  |  |  | DMSO TTFields(+) IR(-) *vs.* Pem TTFields(+) IR(-) | | 0.9943 | ns | |
|  |  |  | Pem TTFields(-) IR(-) *vs.* Pem TTFields(+) IR(-) | | 0.9999 | ns | |
|  |  | 1h (IR+) | DMSO TTFields(-) IR(+) *vs.* DMSO TTFields(+) IR(+) | | 0.1661 | | ns |
|  |  |  | DMSO TTFields(-) IR(+) *vs.* Pem TTFields(-) IR(+) | | 0.9968 | | ns |
|  |  |  | DMSO TTFields(-) IR(+) *vs.* Pem TTFields(+) IR(+) | | 0.9987 | | ns |
|  |  |  | DMSO TTFields(+) IR(+) *vs.* Pem TTFields(+) IR(+) | | 0.0769 | | ns |
|  |  |  | Pem TTFields(-) IR(+) *vs.* Pem TTFields(+) IR(+) | | 0.9532 | | ns |
|  |  | 24h IR(+) | DMSO TTFields(-) IR(+) *vs.* DMSO TTFields(+) IR(+) | | 0.4851 | | ns |
|  |  |  | DMSO TTFields(-) IR(+) *vs.* Pem TTFields(-) IR(+) | | >0.9999 | | ns |
|  |  |  | DMSO TTFields(-) IR(+) *vs.* Pem TTFields(+) IR(+) | | 0.6128 | | ns |
|  |  |  | DMSO TTFields(+) IR(+) *vs.* Pem TTFields(+) IR(+) | | >0.9999 | | ns |
|  |  |  | Pem TTFields(-) IR(+) *vs.* Pem TTFields(+) IR(+) | | 0.5370 | | ns |
|  | **Comparison**  *(One-way ANOVA* +  *Tukey’s* multiple comparisons test) | | | **Adjusted P value** | | **Significance** | |
| **Figure 4C** | **GC3** | DMSO TTFields(-) IR(-) *vs.* DMSO TTFields(-) IR(+) | | <0.0001 | | **** | |
|  |  | DMSO TTFields(-) IR(-) *vs.* DMSO TTFields(+) IR(+) | | <0.0001 | | **** | |
|  |  | DMSO TTFields(-) IR(-) *vs.* Pem TTFields(-) IR(+) | | <0.0001 | | **** | |
|  |  | DMSO TTFields(-) IR(-) *vs.* Pem TTFields(+) IR(+) | | <0.0001 | | **** | |
|  |  | DMSO TTFields(-) IR(+) *vs.* DMSO TTFields(+) IR(+) | | 0.8227 | | ns | |
|  |  | DMSO TTFields(-) IR(+) *vs.* Pem TTFields(-) IR(+) | | 0.8968 | | ns | |
|  |  | DMSO TTFields(-) IR(+) *vs.* Pem TTFields(+) IR(+) | | 0.0307 | | * | |
|  |  | DMSO TTFields(+) IR(+) *vs.* Pem TTFields(-) IR(+) | | >0.9999 | | ns | |
|  |  | DMSO TTFields(+) IR(+) *vs.* Pem TTFields(+) IR(+) | | 0.2508 | | ns | |
|  |  | Pem TTFields(-) IR(+) *vs.* Pem TTFields(+) IR(+) | | 0.2410 | | ns | |
|  | **GC4** | DMSO TTFields(-) IR(-) *vs.* DMSO TTFields(-) IR(+) | | <0.0001 | | **** | |
|  |  | DMSO TTFields(-) IR(-) *vs.* DMSO TTFields(+) IR(+) | | <0.0001 | | **** | |
|  |  | DMSO TTFields(-) IR(-) *vs.* Pem TTFields(-) IR(+) | | <0.0001 | | **** | |
|  |  | DMSO TTFields(-) IR(-) *vs.* Pem TTFields(+) IR(+) | | <0.0001 | | **** | |
|  |  | DMSO TTFields(-) IR(+) *vs.* DMSO TTFields(+) IR(+) | | 0.5220 | | ns | |
|  |  | DMSO TTFields(-) IR(+) *vs.* Pem TTFields(-) IR(+) | | 0.0181 | | * | |
|  |  | DMSO TTFields(-) IR(+) *vs.* Pem TTFields(+) IR(+) | | 0.0002 | | *** | |
|  |  | DMSO TTFields(+) IR(+) *vs.* Pem TTFields(-) IR(+) | | 0.3932 | | ns | |
|  |  | DMSO TTFields(+) IR(+) *vs.* Pem TTFields(+) IR(+) | | 0.0126 | | * | |
|  |  | Pem TTFields(-) IR(+) *vs.* Pem TTFields(+) IR(+) | | 0.4305 | | ns | |

**Supplementary Table 10 :** Summary statistics of data presented in Figure 4. *(*p<0.05 ; **p<0.01 ; ***p<0.001 ; ****p<0.0001 ; ns : not-significant).*
