# Supplementary material for: FGFR inhibition as a new therapeutic strategy to sensitize glioblastoma stem cells to tumor treating fields
Source: Cell Death Discov. 2025 Jun 4;11:265. doi: 10.1038/s41420-025-02542-5 (PMC12137614; doi:10.1038/s41420-025-02542-5)
Supplement: Supplementary file 12 — Supplementary Methods [file 41420_2025_2542_MOESM12_ESM.pdf]

Supplementary Methods:

Clonogenic assay with IR followed by TTFields.

Dissociated GSC were irradiated and within 1hour after IR, cells were seeded into Inovitro™ dishes and treated with TTFields for 72hours at 200kHz. After TTFields application cells were dissociated and 500cells/well were plated in complete medium in 96-wells plates. After 7days of incubation at 37°C and 5% CO_2, whole wells were imaged using an Operetta CLS Imaging system (Perkin Elmer, Waltham, MA, USA) and the number of sphere per well was quantified.

Flow Cytometry (*FGFR1 expression analysis)*

After 72hours of TTFields application, cells were collected and permeabilized using Cytofix/cytoperm kit (BD Biosciences, Le Pont de Claix, France) for 30min in the dark at 4°C. After washing, cells were incubated for 30min at 4°C in PBS (Sigma-Aldrich, Saint-Quentin Fallavier, France) containing 10% SVF (Dutscher, Bernolsheim, France) to avoid unspecific binding and then, incubated for 1hour in the dark at 4°C with FGFR1 PE-conjugated primary antibody (#12777, Cell Signaling, Danvers, MA, USA) or Rabbit (DAE1) mAB IgG XP® Isotype Control (#5742, Cell Signaling, Danvers, MA, USA).

After a wash in PBS (Sigma-Aldrich, Saint-Quentin Fallavier, France), fluorescent signal was measured using a MACSQuant VYB cytometer (Miltenyi Biotec, Bergisch Gladbach, Germany). For each sample, a total of at least 10 000events was recorded and data were analysed using FlowJo™ v10.9 Software (BD Life Sciences, Franklin Lakes, NJ, USA). To evaluate the marker expression, Specific Fluorescence Index (SFI) was determined using the Geometric mean fluorescence intensity (Geomean). The SFI was calculated as previously described with the formula SFI = (Geomean antibody – Geomean isotype control) / Geomean isotype control (73,75).

Immunofluorescence (*γH2AX staining)*

Neurospheres were dissociated and plated on glass coverslips (Knittel Glasbearbeitungs, Braunschweig, Germany) coated with laminin (1.5µg/cm^2, Sigma-Aldrich, Saint-Quentin Fallavier, France). After 24hours at 37°C and 5% CO_2, coverslips were transferred into Inovitro™ ceramic dishes containing complete medium supplemented with Pem or DMSO and TTFields treatment was applied for 72hours. Coverslips were irradiated and harvested 1hour and 24hours after IR. Coverslips were washed in PBS (Sigma-Aldrich, Saint-Quentin Fallavier, France) and fixed in Parafomaldehyde 4% (Santa Cruz Biotechnology, Dallas, TX, USA) for 10min at room temperature. After washes, cells were blocked in PBS 1X (Sigma-Aldrich, Saint-Quentin Fallavier, France) containing 5% BSA (Euromedex, Souffelweyersheim, France) and 0.1% Triton X100 (Sigma-Aldrich, Saint-Quentin Fallavier, France) at room temperature for 1hour. Primary antibody against phosphorylated (Ser139) H2A.X (BioLegend, San Diego, CA, USA) was then incubated in PBS 1X, 3% BSA over night at 4°C, followed by washes with PBS. Coverslips were then incubated with secondary antibody Alexa Fluor® 488 conjugate (#4408, Cell Signaling, Danvers, MA, USA) for 1hour at room temperature in the dark. Nucleus were stained with DAPI (0.2 µg/ml, Sigma-Aldrich, Saint-Quentin Fallavier, France) for 10 minutes at room temperature in the dark. After washes in PBS the fluorescence was visualized using a LSM 880 Fast Airyscan confocal microscope (Zeiss, Oberkochen, Germany) with a 63x NA 1.4 oil-immersion objective.

Quantification of γH2AX foci by image analysis

In order to quantify the evolution of γH2AX signal (green staining) among the nucleus (blue staining, DAPI), we employed a simple Python pipeline based on the python scikit-image library. We begin by separating the nuclei from the background for analysis. Using the OTSU thresholding method in the HSV image space, we obtain a noisy mask, which is further post-processed by removing small objects (smaller than 300 pixels in our case), eroding the mask to cancel out thresholding artefacts, and keeping only the convex hull of every mask. Finally, we remove masks which intersect with the border of the image in order to work with complete nuclei only.

Once we obtain a segmentation mask indicating the regions of interest on the image, we can quantify the pixel ratio evolution for a given experimental condition. Considering that nuclei have different surfaces, we compute a metric which could be interpreted independently from the nucleus size. Furthermore, given that every pixel from the nuclei has a proportion of green and blue channel components, we propose to classify a pixel as marked if the ”green” intensity of the pixel is larger than the ”blue” one. With this in mind, for a given image, we enumerate the number of nuclei detected, and for each mask Ni, we count the number of pixels classified as ”green” G_{Ni} and the total number of pixels contained in the mask T_{Ni} (*i.e* the surface of the nucleus). By taking the ratio of these quantities, we can evaluate the proportion of γH2AX foci contained in a nucleus. We report all three of these measures for every nucleus of an image, and these are then averaged over every condition tested. The complete pipeline is illustrated in **Supplementary Figure 7**.
