# Supplementary material for: FGFR inhibition as a new therapeutic strategy to sensitize glioblastoma stem cells to tumor treating fields
Source: Cell Death Discov. 2025 Jun 4;11:265. doi: 10.1038/s41420-025-02542-5 (PMC12137614; doi:10.1038/s41420-025-02542-5)

<u>Western Blot Full size 1</u> (Figure 2B)

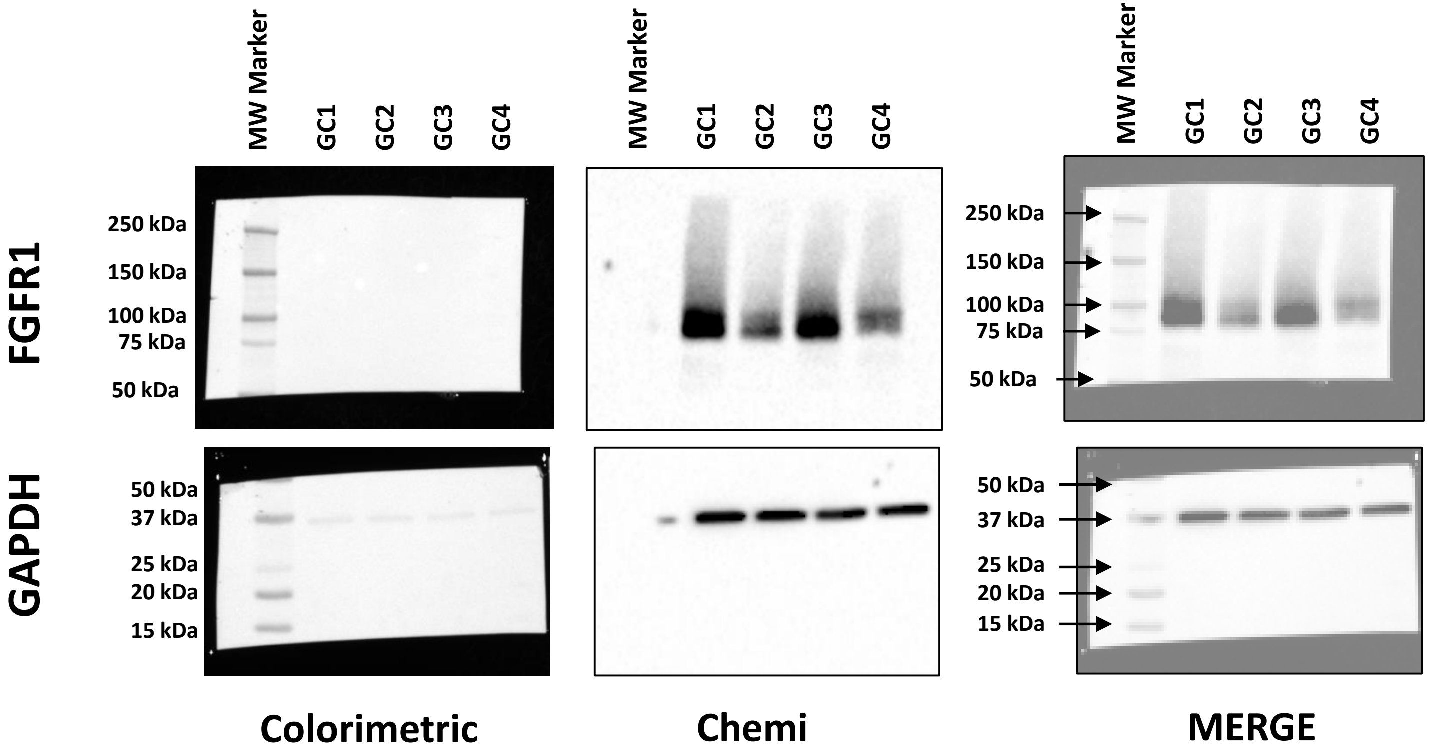

Western Blot Full size 2 (Figure 2B)

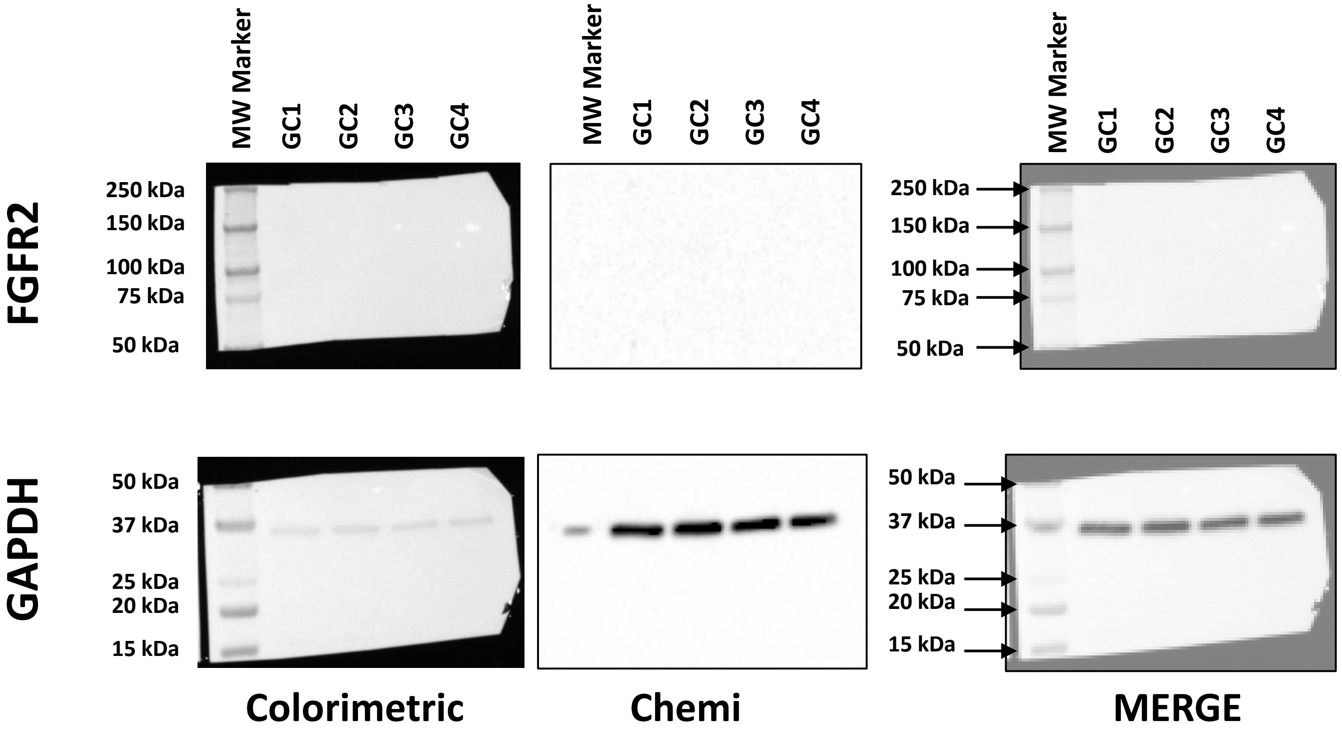

<u>Western Blot Full size 3</u> (Figure 2B)

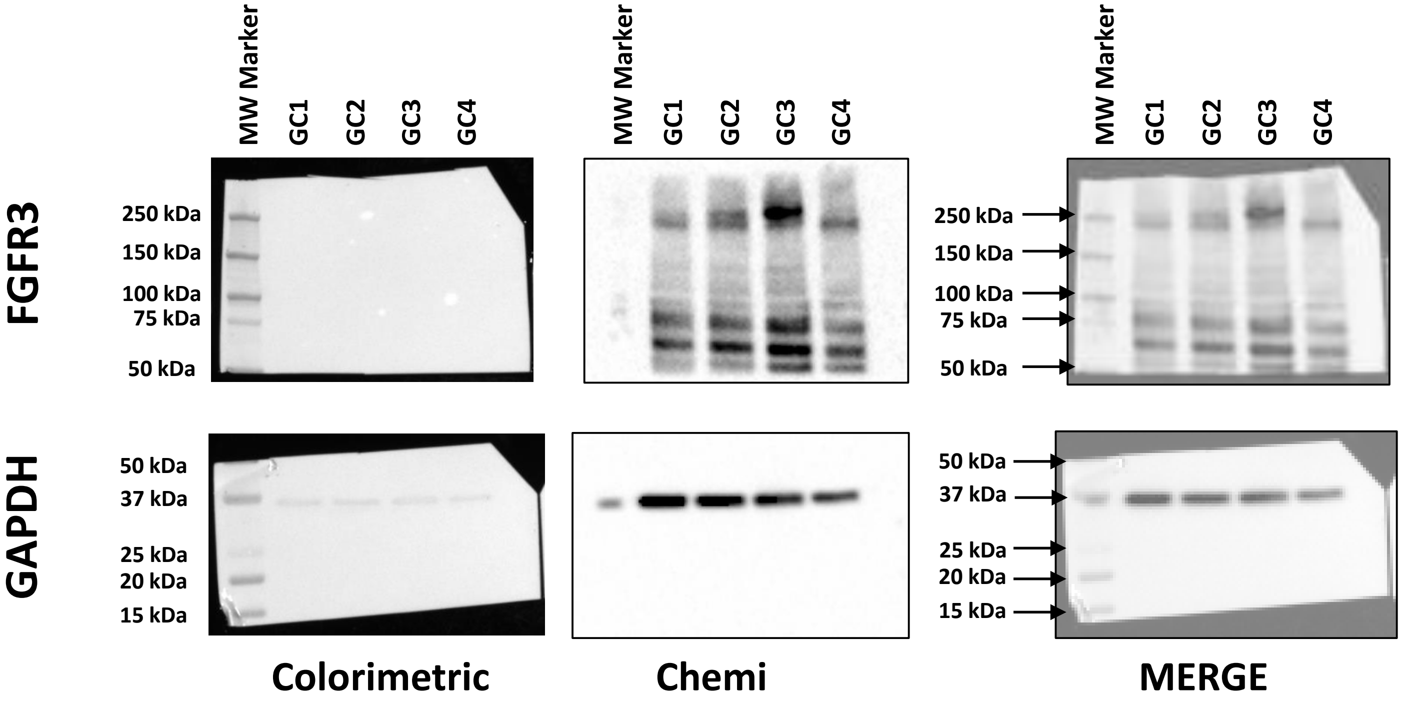

Western Blot Full size 4 (Figure 2B)

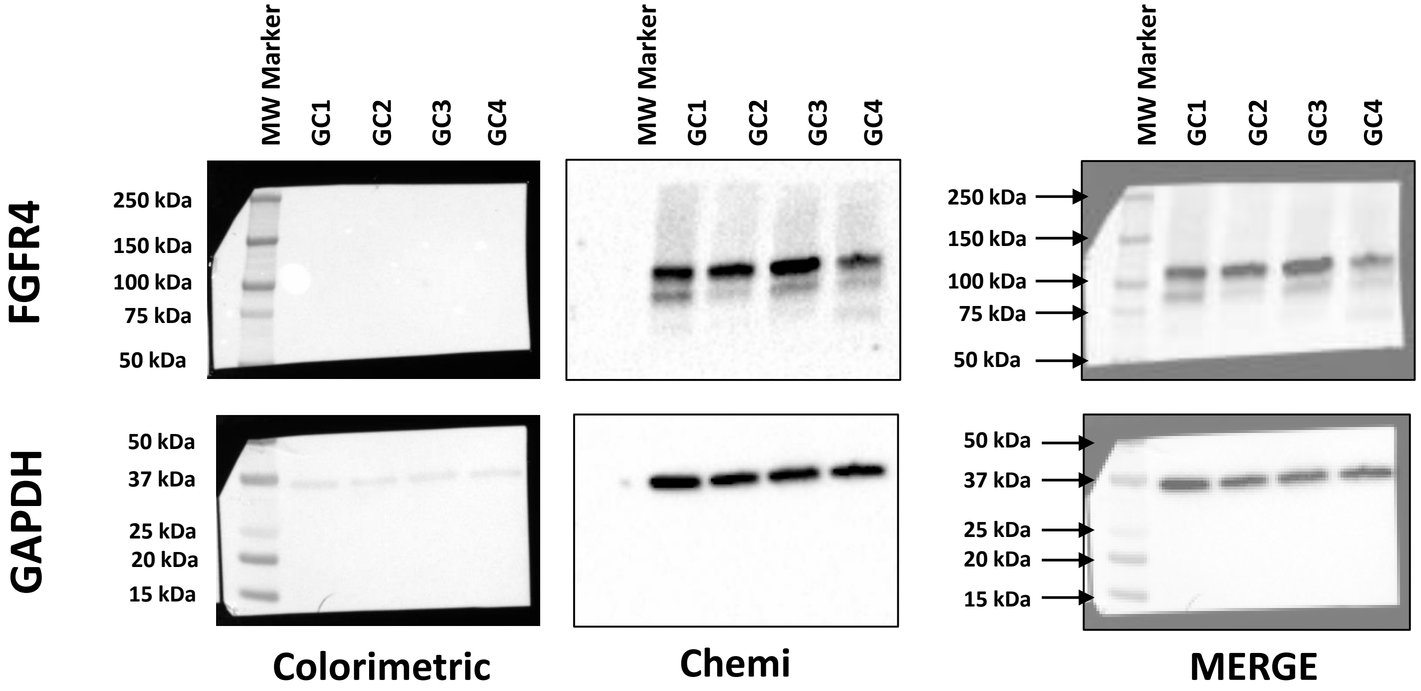

Western Blot Full size 5 (Figure 2D)

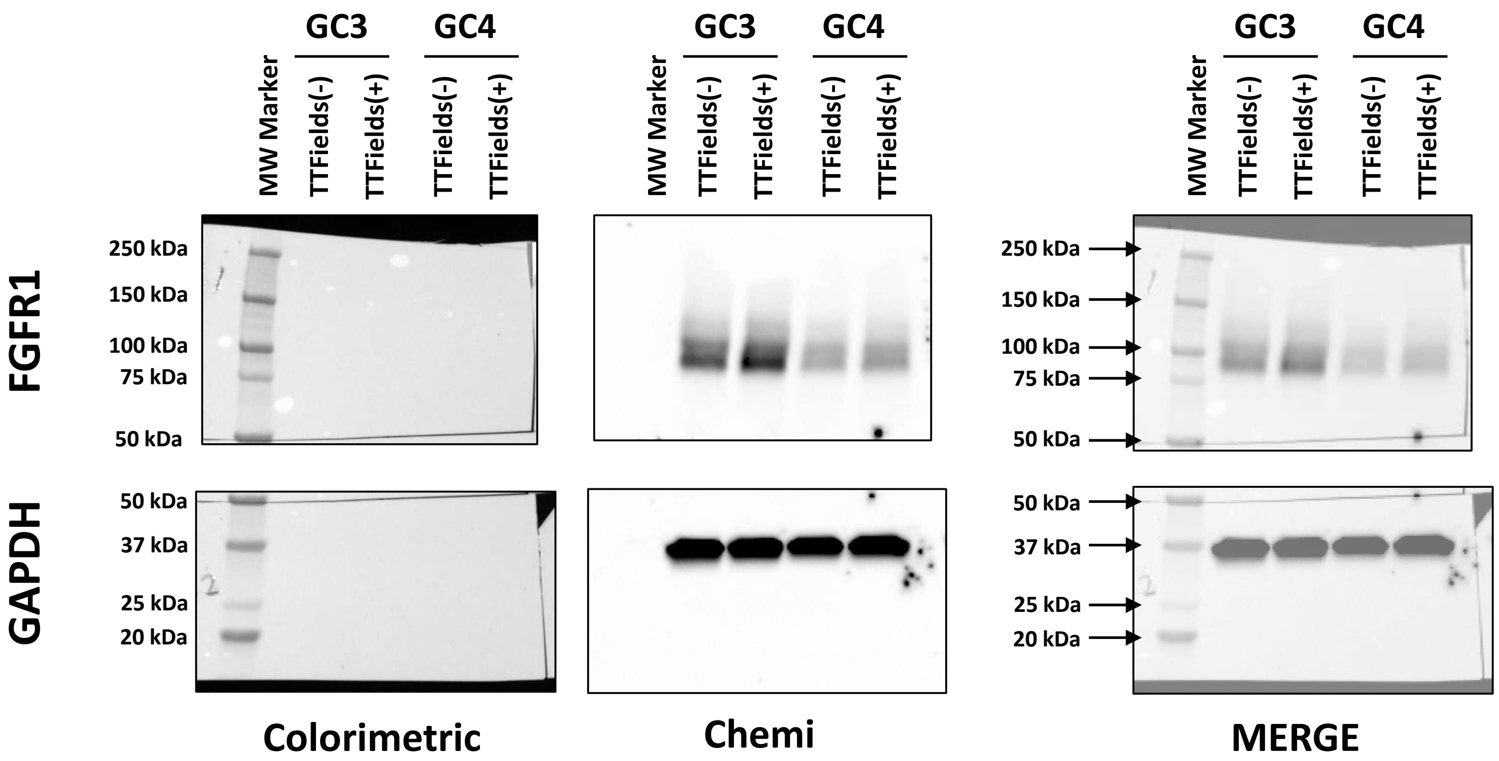

Western Blot Full size 6 (Figure 2D)

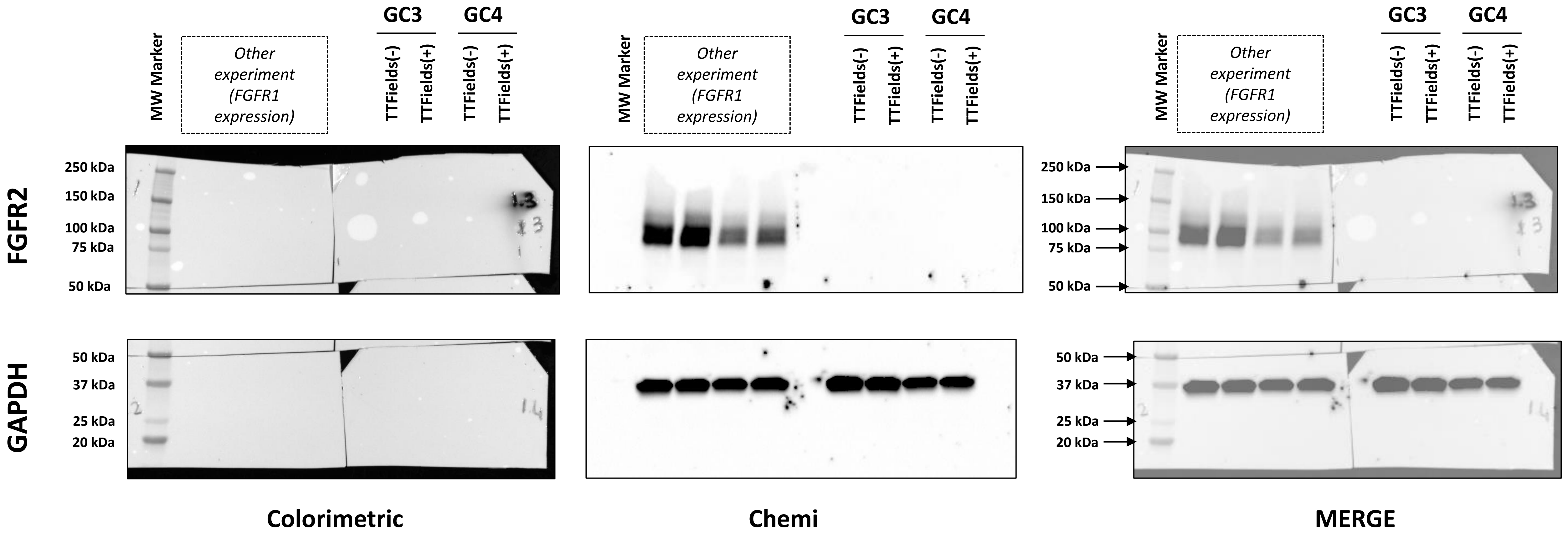

Western Blot Full size 7 (Figure 2D)

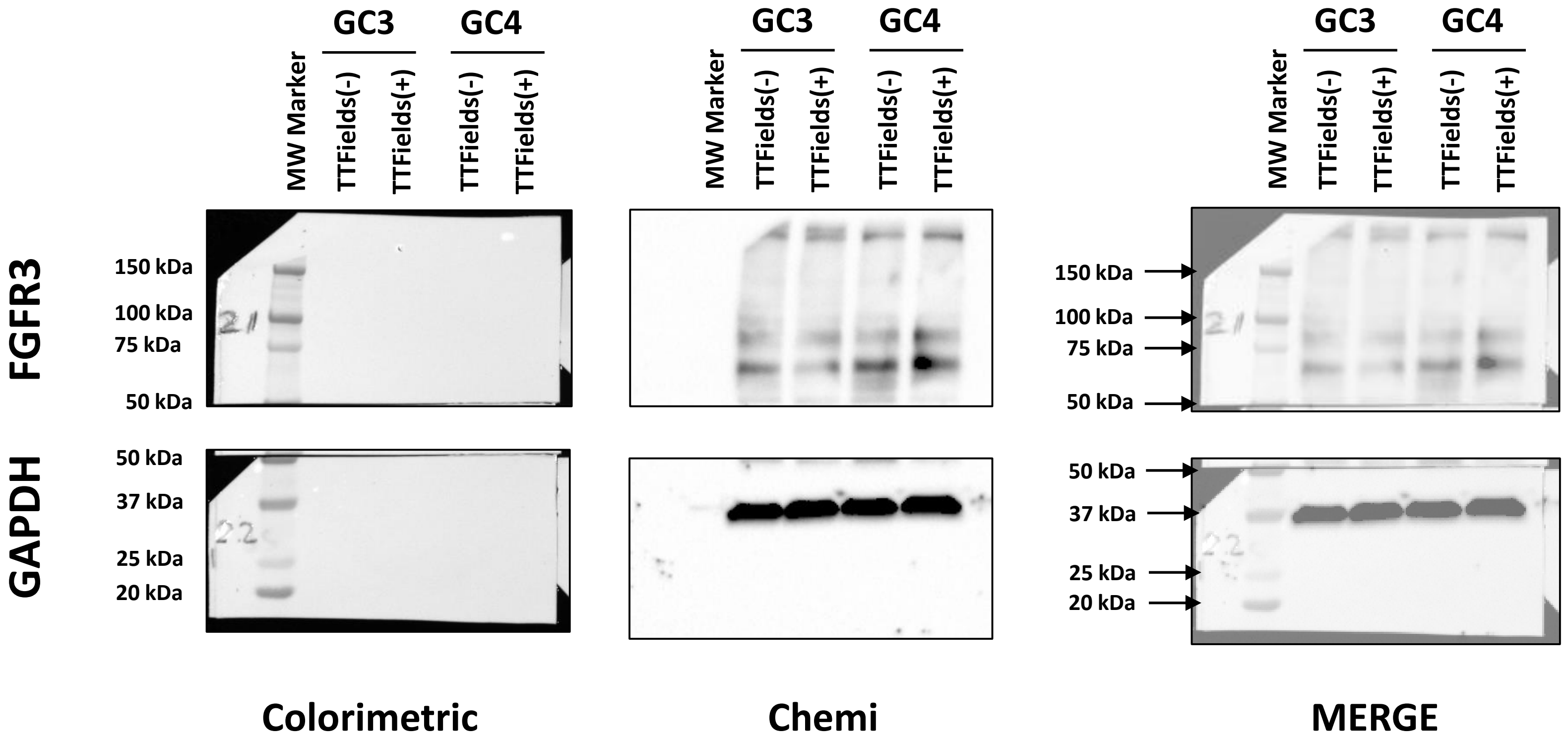

Western Blot Full size 8 (Figure 2D)

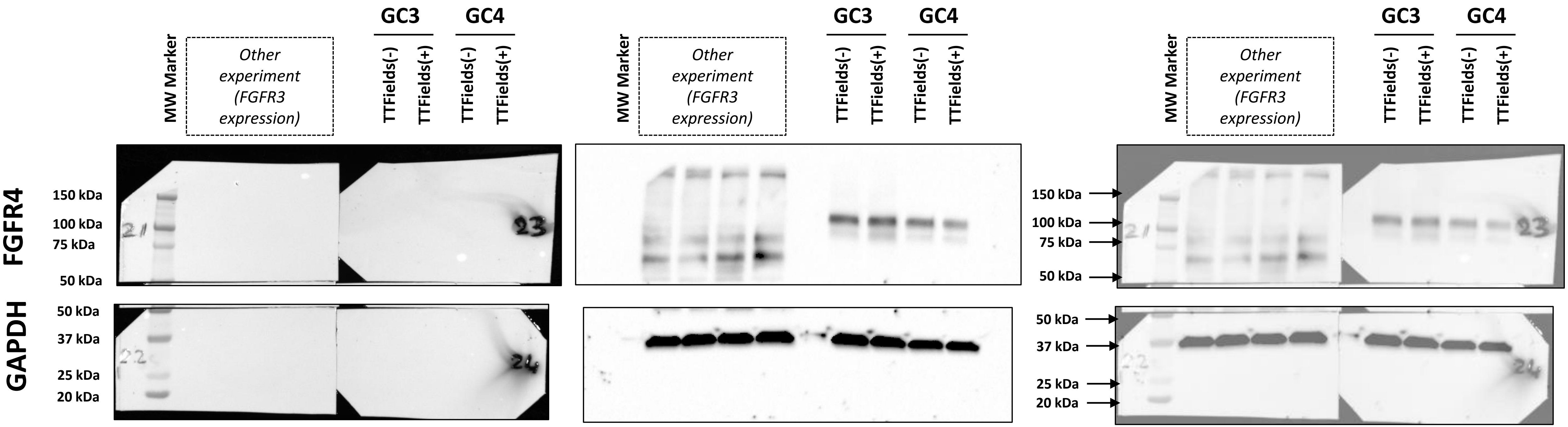

Western Blot Full size 9 (Figure 3A)

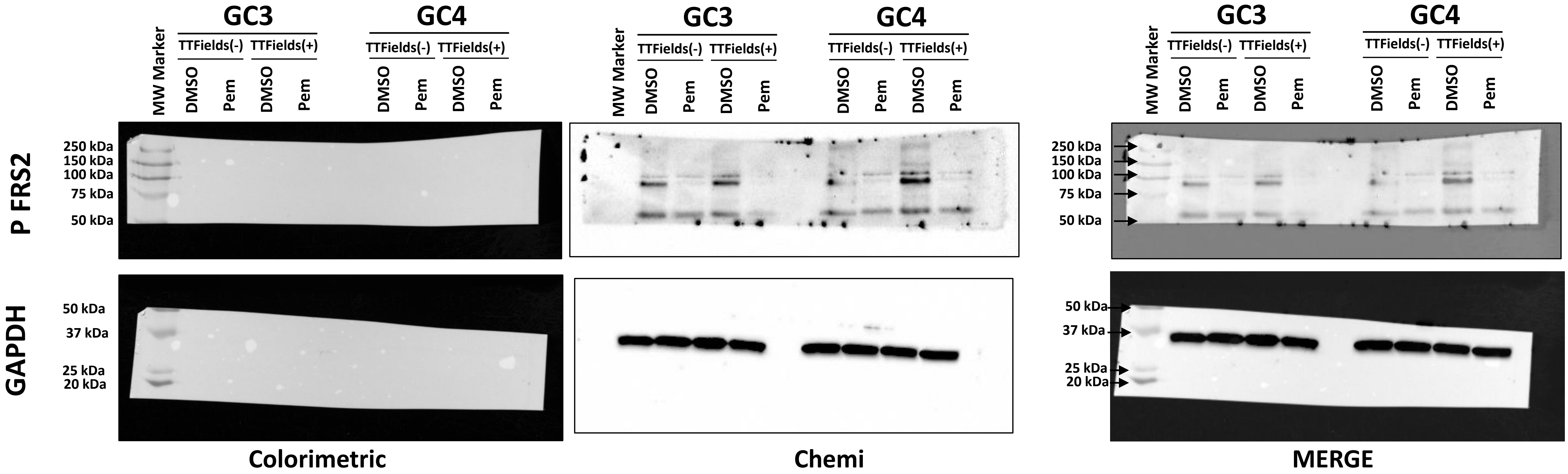

Supplement: Supplementary file 13 — Original western blots [file 41420_2025_2542_MOESM13_ESM.pdf]
